# Targeting virulence: salmochelin modification tunes the antibacterial activity spectrum of β-lactams for pathogen-selective killing of *Escherichia coli* †
†Electronic supplementary information (ESI) available: Tables of bacterial strains employed in this study, iron content of the antimicrobial activity medium, characterization of GlcEnt–Amp/Amx **7–10**, GlcEnt-PEG_3_-N_3_ **12–13**, and BLAST search for *iroN* sequence. Figures of HPLC traces of MceC- and IroB-catalyzed glucosylation of Ent-PEG_3_-N_3_ **11**, optical absorption spectra of GlcEnt–Amp/Amx **7–10**, additional antimicrobial activity assays, time-kill kinetics, competition assays for FepA and IroN recognition, mixed-species antimicrobial activity assays, Lcn2 effect on antibacterial activity of GlcEnt–Amp/Amx **7–10**, and cytotoxicity assays against T84 cells. See DOI: 10.1039/c5sc00962f



**DOI:** 10.1039/c5sc00962f

**Published:** 2015-05-22

**Authors:** Phoom Chairatana, Tengfei Zheng, Elizabeth M. Nolan

**Affiliations:** a Department of Chemistry , Massachusetts Institute of Technology , Cambridge , MA 02139 , USA . Email: lnolan@mit.edu ; Fax: +1-617-324-0505 ; Tel: +1-617-452-2495

## Abstract

New antibiotics are required to treat bacterial infections and counteract the emergence of antibiotic resistance. Pathogen-specific antibiotics have several advantages over broad-spectrum drugs, which include minimal perturbation to the commensal microbiota. We present a strategy for targeting antibiotics to bacterial pathogens that utilises the salmochelin-mediated iron uptake machinery of Gram-negative *Escherichia coli*. Salmochelins are C-glucosylated derivatives of the siderophore enterobactin. The biosynthesis and utilisation of salmochelins are important for virulence because these siderophores allow pathogens to acquire iron and evade the enterobactin-scavenging host-defense protein lipocalin-2. Inspired by the salmochelins, we report the design and chemoenzymatic preparation of glucosylated enterobactin–β-lactam conjugates that harbour the antibiotics ampicillin (Amp) and amoxicillin (Amx), hereafter GlcEnt–Amp/Amx. The GlcEnt scaffolds are based on mono- and diglucosylated Ent where one catechol moiety is functionalized at the C5 position for antibiotic attachment. We demonstrate that GlcEnt–Amp/Amx provide up to 1000-fold enhanced antimicrobial activity against uropathogenic *E. coli* relative to the parent β-lactams. Moreover, GlcEnt–Amp/Amx based on a diglucosylated Ent (DGE) platform selectively kill uropathogenic *E. coli* that express the salmochelin receptor IroN in the presence of non-pathogenic *E. coli* and other bacterial strains that include the commensal microbe *Lactobacillus rhamnosus* GG. Moreover, GlcEnt–Amp/Amx evade the host-defense protein lipocalin-2, and exhibit low toxicity to mammalian cells. Our work establishes that siderophore–antibiotic conjugates provide a strategy for targeting virulence, narrowing the activity spectrum of antibiotics in clinical use, and achieving selective delivery of antibacterial cargos to pathogenic bacteria on the basis of siderophore receptor expression.

## Abstract 2

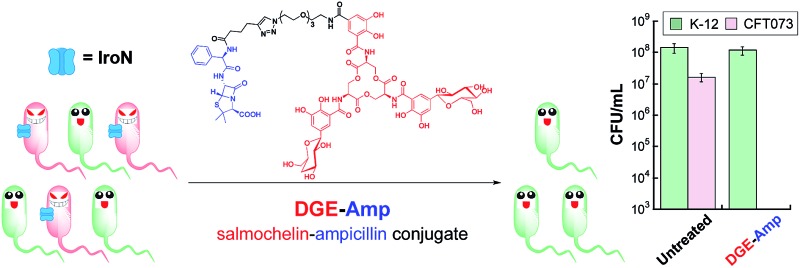
New antibiotics are required to treat bacterial infections and counteract the emergence of antibiotic resistance.

## Introduction

Bacterial infections, the rise in antibacterial resistance in hospital and community settings, and the paucity of new antibiotics in the current drug pipeline create a worldwide public health crisis.^1,2^ New strategies to diagnose and treat bacterial infections as well as counteract the emergence and spread of antibiotic resistance in bacterial pathogens are urgently needed to reduce morbidity and mortality, as well as the economic burden, caused by these infections.^3,4^ The discovery of narrow-spectrum antibiotics that target select pathogens is one important and necessary facet of this large and complex problem.^2,5,6^ Pathogen-specific antibiotics that minimally perturb the normal microbial flora are expected to reduce the likelihood of colonisation by pathogenic and drug-resistant microbes during or after antibiotic treatment, and prevent life-threatening secondary infections such as those caused by *Clostridium difficile*.^5,7^ Moreover, the availability of narrow-spectrum antibiotics, coupled with rapid diagnostics, is expected to reduce the use of broad-spectrum therapeutics and thereby slow down the evolution of drug resistance.^2,5,7^ Among current and emerging microbial threats, Gram-negative bacteria, including pathogenic *Escherichia coli*, *Klebsiella pneumoniae*, *Acinetobacter baumannii*, and *Salmonella* spp., pose a challenge for antibiotic drug discovery.^1,2,8^ These strains have an outer membrane that serves as a permeability barrier and prevents the cellular entry of many antibiotics.^9^ In this work, we report a stealth antibiotic delivery strategy that overcomes the outer membrane permeability barrier of Gram-negative *E. coli* and targets pathogenicity by hijacking the iron import machinery utilised by virulent strains during colonisation in the mammalian host.

Iron is an essential nutrient for almost all bacterial pathogens.^10,11^ Because iron exhibits low solubility in aqueous solutions at physiological pH and enables Fenton chemistry, the levels of “free” iron in mammals (*ca.* 10^–24^ M in serum)^12^ are tightly regulated by homeostatic mechanisms, which include the expression of the iron transport and storage proteins transferrin and ferritin.^13^ Most bacterial pathogens require micromolar concentrations of iron to colonise and cause disease, and bacterial iron acquisition machineries contribute to virulence.^10,14^

**Fig. 1:**
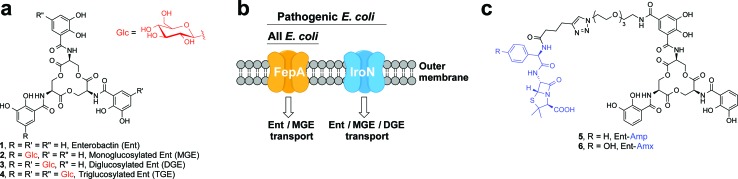
Siderophores and outer membrane siderophore receptors relevant to this work. (a) Chemical structures of enterobactin **1**, and its glucosylated derivatives **2–4**. (b) A cartoon representation of the outer membrane receptors FepA and IroN considered in this work. (c) Chemical structures of Ent–Amp/Amx **5** and **6**.

One way that bacteria scavenge iron in the host environment is to biosynthesize and export siderophores, secondary metabolites that chelate Fe(iii) with high affinity.^15^ The ferric siderophores are recognised and transported into the cell by dedicated uptake machinery. In this work, we consider the catecholate siderophore enterobactin **1** (Ent, Fig. 1a), its glucosylated congeners **2–4** (GlcEnt, Fig. 1a), and the outer membrane receptors for these iron chelators. Ent is biosynthesized by all *E. coli* and the ferric complex is transported across the outer membrane by the TonB–ExbB–ExbD-dependent outer membrane receptor FepA (Fig. 1b).^12^ In addition to Ent, many pathogenic *E. coli* as well as *Salmonella* spp. biosynthesize salmochelins, C-glucosylated derivatives of Ent (Fig. 1a).^16^ The *iroA* gene cluster (*iroBCDEN*)^14,17,18^ encodes enzymes that tailor the Ent scaffold to provide the salmochelins (IroBDE), and proteins for salmochelin transport (IroCN). Expression of genes encoded by the *iroA* locus contributes to virulence by providing Gram-negative pathogens with additional iron acquisition machinery and enabling the pathogens to overcome the host innate immune response.^19,20^ In the battle against such invading pathogens, the mammalian host mounts a metal-withholding response and secretes lipocalin-2 (Lcn2), a 22 kDa antimicrobial protein that captures ferric Ent.^19,21,22^ Gram-negative pathogens that utilise salmochelins for iron acquisition readily evade this innate immune mechanism because the salmochelins cannot be sequestered by Lcn2.^19^

Because bacteria utilise siderophores to acquire nutrient iron during infection, these molecules, as well as the corresponding biosynthetic and transport machineries, provide opportunities for antibiotic development.^10,11,23–27^ The notion of using siderophores or siderophore mimics to deliver antibacterial cargo into bacterial cells has garnered attention over several decades.^28–44^ Our approach to siderophore-based targeting focuses on harnessing native siderophore platforms used by pathogens in the human host for cargo delivery, and we seek to modify these scaffolds in ways that minimally perturb iron binding and receptor recognition. We have designed and utilised a monofunctionalized Ent platform to assemble a variety of Ent–cargo conjugates, and we reported that the Ent uptake machinery (FepABCDG) provides a means to transport small-molecule cargo, including antibiotics in clinical use, into *E. coli*.^44,45^ For instance, the Ent–β-lactam conjugates **5** and **6** (Fig. 1c) target and kill *E. coli* expressing FepA.^44^ Because all *E. coli* use Ent for iron acquisition, the Ent–β-lactam conjugates target and kill both non-pathogenic and pathogenic *E. coli* strains. Some *E. coli* are commensal microbes, comprising <1% of the total microbial community in the human gut, that biosynthesize vitamin K that is needed by the host.^46,47^ Thus, the ability to target pathogenic *E. coli* has utility for minimally perturbing the normal flora. Inspired by prior investigations of native siderophore transport,^16,48^ we hypothesised that salmochelin–antibiotic conjugates will be specifically recognised by IroN, the outer membrane receptor for the salmochelins, and afford a strategy for overcoming the outer membrane permeability barrier, achieving narrow-spectrum antibacterial activity against pathogenic *E. coli*, and evading capture by Lcn2.

In this work, we report the design and chemoenzymatic preparation of siderophore–β-lactam conjugates based on salmochelin platforms, and demonstrate targeting of β-lactam antibiotics to pathogenic *E. coli* that harbour the *iroA* gene cluster and express IroN. Salmochelin-inspired GlcEnt–β-lactam conjugates based on the diglucosylated Ent (DGE, Fig. 1a) platform provide selective antibacterial activity against pathogenic *E. coli* and up to 1000-fold enhanced potency relative to the parent β-lactam antibiotics. Moreover, the salmochelin–inspired conjugates remain antibacterial in the presence of Lcn2. These investigations establish a chemoenzymatic route to functionalized salmochelins and provide a new approach for transforming a broad-spectrum antibiotic in clinical use into a narrow-spectrum therapeutic that targets microbial pathogens on the basis of siderophore receptor expression.

## Results and discussion

### Design and syntheses of GlcEnt–β-lactam conjugates

We present a family of salmochelin–inspired GlcEnt–β-lactam conjugates **7–10** that exhibit ampicillin (Amp) or amoxicillin (Amx) attached to either monoglucosylated Ent (MGE, **2**) or diglucosylated Ent (DGE, **3**) by a stable polyethylene glycol (PEG_3_) linker. The design of the GlcEnt–β-lactam conjugates **7–10** builds upon Ent–Amp/Amx **5** and **6** (Fig. 1C).^44^ These conjugates are based on a monosubstituted Ent platform where one catechol moiety is modified at the C5 position for cargo attachment. We sought to install glucose moieties at the C5 position of one or both of the unfunctionalized catechol rings to afford MGE–Amp/Amx **7** and **8** and DGE–Amp/Amx **9** and **10**, respectively. Although the total chemical syntheses of salmochelins have been reported, nine steps are required to achieve the requisite glucosylated 2,3-dihydroxybenzoic acid building block.^49^ We therefore established a chemoenzymatic approach that employs enzymes involved in salmochelin biosynthesis, which affords the desired glucosylated conjugates and requires only one additional step compared to the reported preparation of Ent–Amp/Amx.

**Scheme 1:**
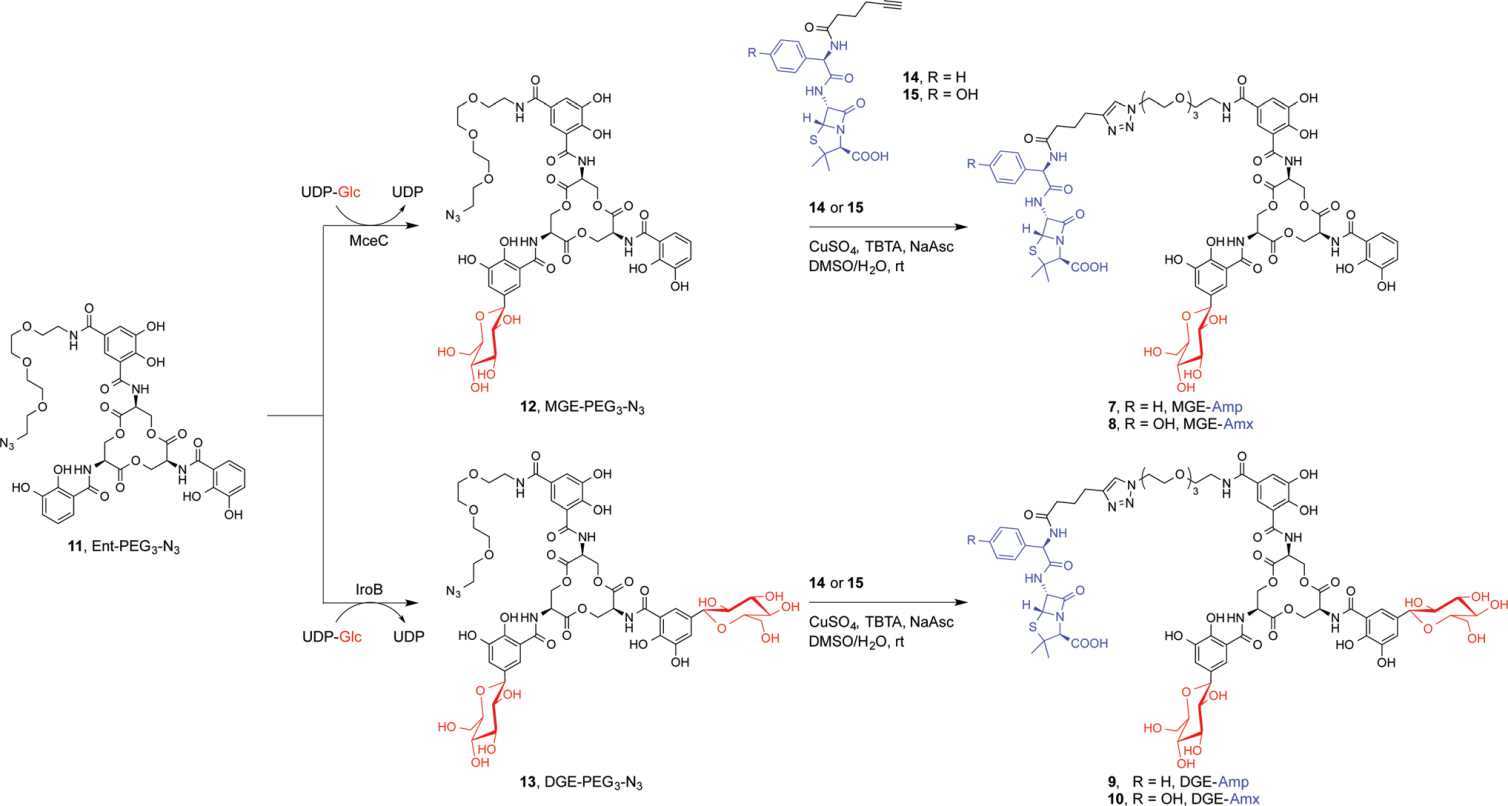
Chemoenzymatic syntheses of GlcEnt–Amp/Amx **7–10**. The synthetic route consists of MceC- or IroB-catalysed glucosylation of Ent-PGE_3_-N_3_ **11** followed by a copper-catalysed click reaction to achieve the GlcEnt–β-lactam conjugates **7–10**. We abbreviate the siderophore family **1–4** (Fig. 1a) as (Glc)Ent and the siderophore–β-lactam conjugates **5–10** as (Glc)Ent–Amp/Amx.

IroB and MceC are C-glucosyltransferases that catalyse C-glucosylation of Ent at the C5 positions of the catechol rings. MceC is encoded by the MccE492 gene cluster of *K. pneumoniae* RYC492, and has 75% amino acid sequence identity with IroB.^50^ IroB catalyses up to three C-glucosylation events, affording MGE, DGE and TGE as products (Fig. 1a).^51^ MceC, in contrast, produces only MGE and DGE.^52^ On the basis of these observations, we hypothesised that both IroB and MceC would accept monofunctionalized Ent as a substrate, providing a preparative route to **7–10**. Initial activity assays where either IroB or MceC was incubated with Ent-PEG_3_-N_3_ **11**, UDP-Glc, and Mg(ii) revealed that both enzymes accept Ent-PEG_3_-N_3_ **11** as a substrate and afford MGE-PEG_3_-N_3_ **12** and DGE-PEG_3_-N_3_ **13** as products (Fig. S1 and S2†). Accumulation of **12** was observed in the MceC-catalysed reactions, whereas **13** accumulated in reactions catalysed by IroB. When Ent–Amp/Amx **5** and **6** were employed as substrates, complex product mixtures were obtained. LC/MS analysis of the mixtures revealed the desired products as well as multiple byproducts, including products of β-lactam decomposition. We therefore performed large-scale C-glucosylation reactions employing Ent-PEG_3_-N_3_ **11** as a substrate to afford milligram quantities of MGE-PEG_3_-N_3_ **12**, and DGE-PEG_3_-N_3_ **13** (Scheme 1). We subsequently employed copper-catalysed azide/alkyne cycloaddition to install the β-lactam moieties (Scheme 1).^44^ This route achieved the mono- and diglucosylated conjugates **7–10** in high purity and in yields of 26–59% from **7** following HPLC purification. As expected, the GlcEnt–β-lactam conjugates **7–10** bind iron.^53^ Each Fe(iii) complex exhibits a broad absorption band (*ca.* 400–700 nm, MeOH) characteristic of ferric Ent and its derivatives (Fig. S3†).^44,45,54^

### DGE–β-lactam conjugates target pathogenic *E. coli* expressing IroN

To evaluate whether GlcEnt–Amp/Amx **7–10** target pathogenic *E. coli* expressing IroN, we compared the antibacterial activities of the parent antibiotics Amp/Amx, Ent–Amp/Amx **5** and **6**, and GlcEnt–Amp/Amx **7–10**. We selected five *E. coli* strains on the basis of siderophore receptor expression (Table S1†). *E. coli* CFT073 ^55^ and UTI89 ^56^ harbour the *iroA* gene cluster, biosynthesize and utilise salmochelins for iron acquisition in the host, and cause urinary tract infections.^57,58^ In contrast, *E. coli* H9049 is a clinical isolate that does not have the *iroA* cluster.^22^ *E. coli* K-12 ^59^ and *E. coli* B ^60^ are non-pathogenic laboratory strains that also lack the *iroA* cluster. To ascertain the effect of iron limitation on antibacterial activity, we performed antibacterial activity assays in the absence or presence of the metal-ion chelator 2,2′-dipyridyl (DP, 200 μM). This concentration of DP inhibits *E. coli* growth (Fig. S4†). These assays revealed that DGE–Amp/Amx **9** and **10** target pathogenic *E. coli* that express IroN.

**Fig. 2:**
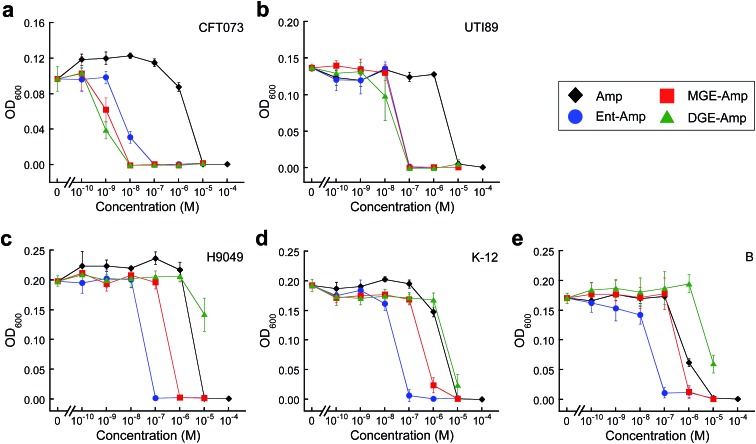
Antibacterial activity of (Glc)Ent–Amp against five *E. coli* strains. (a)–(e) Antibacterial activity of (Glc)Ent–Amp **5**/**7**/**9** against (a) uropathogenic *E. coli* CFT073, (b) uropathogenic *E. coli* UTI89, (c) non-pathogenic clinical isolate *E. coli* H9049, (d) laboratory strain *E. coli* K-12, (e) laboratory strain *E. coli* B. All assays were performed in 50% MHB medium supplemented with 200 μM DP (*t* = 19 h, *T* = 30 °C) (mean ± standard deviation, *n* ≥ 3). The data for (Glc)Ent–Amx **6**/**8**/**10** and for the assays performed in the absence of DP are presented in Fig. S5–S9.†

Amp/Amx exhibit minimum inhibitory concentration (MIC) values of 10 μM against the five *E. coli* strains (±DP, Fig. 2 and S5–S9†). Under conditions of iron limitation, Ent–Amp/Amx provide 100- to 1000-fold enhanced activity against all five strains (50% MHB, +DP). These results are in agreement with our prior studies of Ent–Amp/Amx killing of *E. coli*.^44^ Glucosylation affords strain-dependent antimicrobial activity that correlates with IroN expression (Fig. 2a and b, S5 and S6†). Like Ent–Amp/Amx **5** and **6**, GlcEnt–Amp/Amx **7–10** provide 100- and 1000-fold enhanced antimicrobial activity against *E. coli* UTI89 and *E. coli* CFT073, respectively (+DP). The susceptibility of *E. coli* CFT073 to GlcEnt–Amp/Amx remains enhanced in the absence of DP, as observed previously for Ent–Amp/Amx.^44^ The antibacterial activity of GlcEnt–Amp/Amx **7–10** against *E. coli* H9049, K-12, and B is attenuated relative to that of Ent–Amp/Amx **5** and **6** (+DP, Fig. 2c–e and S7–S9†). Moreover, for these non-pathogenic strains, the MIC values of (Glc)Ent–Amp/Amx follow the trend Ent–Amp/Amx < MGE–Amp/Amx < DGE–Amp/Amx. The MGE modification provides enhanced potency relative to Amp/Amx because growth reduction (K-12) or complete growth inhibition (H9049 and B) occurs at 1 μM MGE–Amp/Amx (+DP). In contrast, the DGE–β-lactam conjugates exhibit negligible antibacterial activity against the three strains that lack IroN (MIC > 10 μM). The growth medium contains ≈4 μM iron (Table S2†) and we attribute the growth inhibition observed at 10 μM DGE–Amp/Amx to iron deprivation that results from DGE–Amp/Amx sequestering the iron in the growth medium (Table S2†).

In the antibacterial activity assays described above, we treated the bacterial cultures with the apo conjugates and expected that the siderophore moieties chelate iron from the growth medium, allowing for recognition of the ferric–siderophore complexes by FepA and IroN. We previously reported that preloading of Ent–Amp/Amx with Fe(iii) prior to antibacterial activity assays against *E. coli* K-12 had negligible effect on the MIC value.^44^ Here we report that preloading of MGE–Amp/Amx and DGE–Amp/Amx also has a negligible effect on the growth inhibitory properties (Fig. S10†). This result is expected given that the concentration of iron in the growth medium far exceeds the MIC values obtained for the conjugates under conditions where FepA and IroN are expressed. Lastly, mixtures of unmodified Amp/Amx and (Glc)Ent **1–3** against *E. coli* CFT073 and UTI89 provide the same MIC values as Amp/Amx alone and confirm that the enhanced antibacterial activity of (Glc)Ent–Amp/Amx **5–10** requires the covalent attachment of β-lactams to the siderophore scaffolds (Fig. S11 and S12†).

### Siderophore modification accelerates killing of pathogenic *E. coli* CFT073

**Fig. 3:**
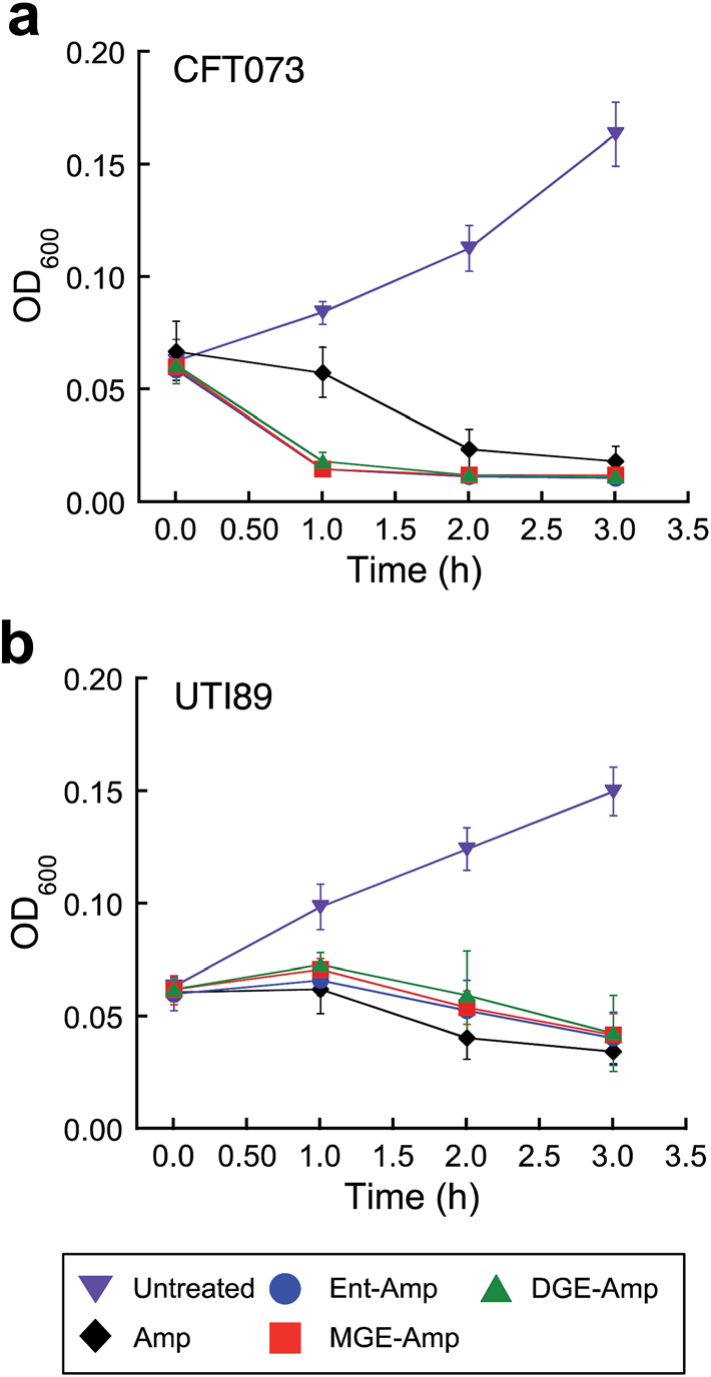
Time-kill kinetics of (Glc)Ent–Amp against *E. coli* CFT073 and UTI89. (a and b) Time-kill kinetics of (Glc)Ent–Amp **5**/**7**/**9** against (a) uropathogenic *E. coli* CFT073 (≈10^8^ CFU mL^–1^) treated with 50 μM Amp or 5 μM (Glc)Ent–Amp **5**/**7**/**9** and (b) uropathogenic *E. coli* UIT89 (≈10^8^ CFU mL^–1^) treated with 50 μM Amp or 50 μM (Glc)Ent–Amp **5**/**7**/**9**. All assays were performed in 50% MHB medium supplemented with 200 μM DP (*T* = 37 °C) (mean ± standard deviation, *n* ≥ 3). The data for (Glc)Ent–Amx **6**/**8**/**10** and for the assays performed in the absence of DP are presented in Fig. S13–S14.†

*E. coli* CFT073 is rapidly killed by conjugates **5–10** (Fig. 3a and S13†). The OD_600_ value of *E. coli* CFT073 culture (10^8^ CFU mL^–1^) treated with 5 μM (Glc)Ent–β-lactam is reduced to almost the baseline value (≈0.04) after 1 h, which corresponds to a ≈2-fold log reduction in CFU mL^–1^, whereas the change in OD_600_ and CFU mL^–1^ for *E. coli* CFT073 treated with 50 μM Amp/Amx is negligible over this time period. In contrast, siderophore modification has negligible effect on the time-kill kinetics observed for *E. coli* UTI89 (Fig. 3b and S14†); the (Glc)Ent–β-lactam conjugates provide similar profiles as observed for Amp/Amx. This result is reminiscent of our prior observations for *E. coli* K-12 where attachment of Ent to Amp/Amx provided only a modest increase in the time-kill kinetics compared to the parent antibiotics.^44^ The origin of this strain-dependence is unclear and warrants further investigation. Nevertheless, these data show that glucosylation of Ent–Amp/Amx does not alter the time-kill kinetics of Ent–Amp/Amx for either *E. coli* CFT073 or UTI89, and (Glc)Ent–Amp/Amx **5–10** kill CFT073 more rapidly than UTI89.

### Siderophore competition supports recognition of (Glc)Ent–β-lactam conjugates by IroN

**Fig. 4:**
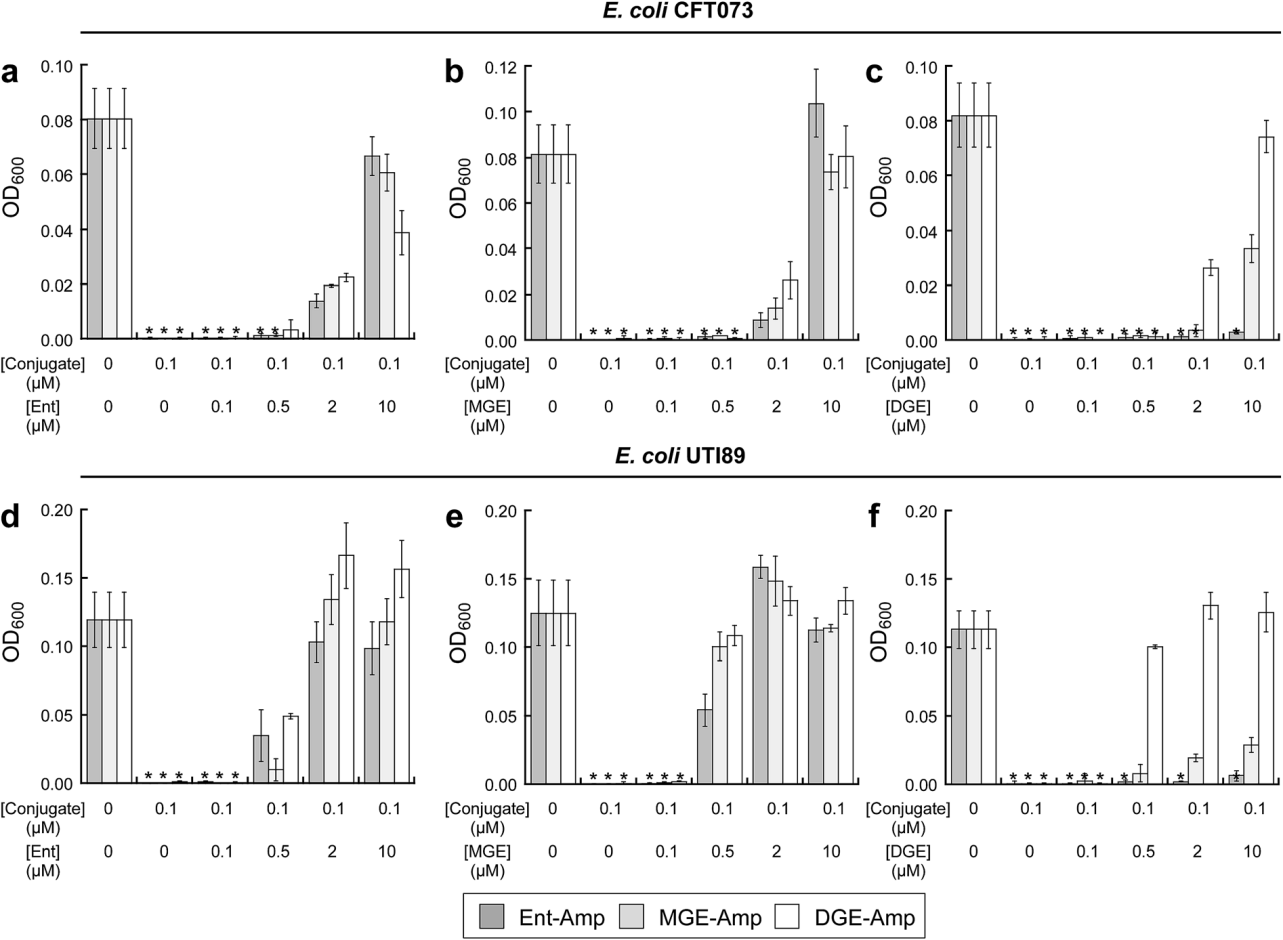
Exogenous (Glc)Ent compete with (Glc)Ent–Amp conjugates for FepA and IroN recognition. (a)–(c) Growth of *E. coli* CFT073 in the presence of 100 nM (Glc)Ent–Amp **5**/**7**/**9** and mixtures of 100 nM (Glc)Ent–Amp **5**/**7**/**9** and 1, 5, 20, or 100 equiv of exogenous (a) Ent **1**, (b) MGE **2**, or (c) DGE **3** in the presence of 200 μM DP. (d)–(f) Growth of *E. coli* UTI89 in the presence of 100 nM (Glc)Ent–Amp **5**/**7**/**9** and mixtures of 100 nM (Glc)Ent–Amp **5**/**7**/**9** and 1, 5, 20, or 100 equiv of exogenous (d) Ent **1**, (e) MGE **2**, or (f) DGE **3** in the presence of 200 μM DP. All assays were performed in 50% MHB medium (*t* = 19 h, *T* = 30 °C) (mean ± standard deviation, *n* = 3). An asterisk indicates OD_600_ < 0.01. The data for (Glc)Ent–Amx **6**/**8**/**10** are presented in Fig. S15.†

To investigate the interactions between (Glc)Ent–β-lactam conjugates **5–10** and the siderophore receptors FepA and IroN of *E. coli* CFT073 and UTI89, we performed modified antimicrobial activity assays where varying concentrations (0–10 μM) of (Glc)Ent **1–3** were combined with 100 nM (Glc)Ent–Amp/Amx **5–10** (Fig. 4 and S15†). These mixtures provide a means to probe competition between exogenous native siderophores and the conjugates for receptor recognition because siderophore uptake of the former molecules results in growth promotion whereas the latter afford growth inhibition. The competition assays establish that Ent and MGE attenuate the antibacterial activity of all (Glc)Ent–β-lactam conjugates **5–10** to varying degrees, whereas DGE only inhibits the activity of the glucosylated congeners **7–10**. Moreover, DGE fully attenuates DGE–Amp/Amx **9–10** but not MGE–Amp/Amx **7** and **8**. These conclusions are drawn from the following observations: (i) a 100-fold molar excess of Ent recovers the growth of *E. coli* CFT073 treated with Ent/MGE–Amp/Amx **5–8** to levels comparable to that of the untreated control (Fig. 4a and S15a†). In contrast, a 100-fold excess of Ent provides only partial growth recovery of *E. coli* CFT073 treated with DGE–Amp/Amx **9** and **10**. (ii) A 100-fold excess of MGE fully recovers the growth of *E. coli* CFT073 treated with (Glc)Ent–Amp/Amx **5–10** (Fig. 4b and S15b†). (iii) A 100-fold molar excess of DGE does not recover the growth of *E. coli* CFT073 treated with Ent–Amp/Amx **5** and **6**, whereas it provides partial and complete growth recovery of *E. coli* CFT073 treated with MGE–Amp/Amx **7** and **8** and DGE–Amp/Amx **9** and **10**, respectively (Fig. 4c and S15c†). In total, this work indicates that FepA recognises and delivers Ent/MGE–Amp/Amx **5–8** but not DGE–Amp/Amx **9** and **10**, whereas IroN binds and transports all conjugates based on the three siderophore scaffolds. Competition assays employing *E. coli* UTI89 afford overall trends that are similar to those observed for *E. coli* CFT073 except that lower concentrations of exogenous siderophores effectively block the antibacterial action of (Glc)Ent–Amp (Fig. 4d–f and S15d–f†).

### GlcEnt–Amp/Amx kill pathogenic *E. coli* in the presence of other microbes that include non-pathogenic *E. coli* and commensal Lactobacilli

**Fig. 5:**
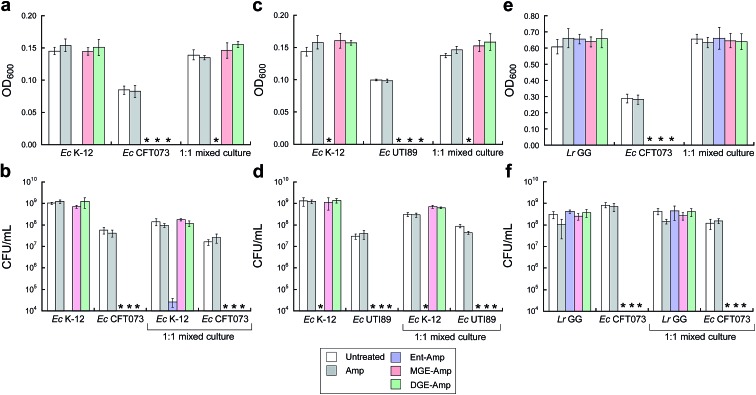
MGE/DGE–Amp selectively kill uropathogenic *E. coli* in the presence of non-pathogenic *E. coli* K-12 and the probiotic *L. rhamnosus* GG. (a and b) Bacterial growth monitored by (a) OD_600_ and (b) CFU mL^–1^ for cultures of *E. coli* K-12 only, CFT073 only, and 1 : 1 K-12/CFT073 mixtures treated with 100 nM Amp or 100 nM (Glc)Ent–Amp **5**/**7**/**9** in the presence of 200 μM DP. (c and d) Bacterial growth monitored by (c) OD_600_ and (d) CFU mL^–1^ for cultures of *E. coli* K-12 only, UTI89 only, and 1 : 1 K-12/UTI89 mixtures treated with 100 nM Amp or 100 nM (Glc)Ent–Amp **5**/**7**/**9** in the presence of 200 μM DP. (e and f) Bacterial growth monitored by (e) OD_600_ and (f) CFU mL^–1^ for cultures of *L. rhamnosus* GG only, *E. coli* CFT073 only, and 1 : 1 *L. rhamnosus* GG/*E. coli* CFT073 mixtures treated with 1 μM Amp or 1 μM (Glc)Ent–Amp **5**/**7**/**9** in the presence of 200 μM DP. All mixed-*E. coli* antimicrobial assays were performed in 50% MHB medium and all mixed-species antimicrobial assays were conducted in 1 : 1 MRS/MHB medium (*t* = 19 h, *T* = 30 °C) (mean ± standard deviation, *n* = 3). An asterisk indicates OD_600_ < 0.01 or no colony formation. The data for (Glc)Ent–Amx **6**/**8**/**10** are presented in Fig. S16.†

To further probe the activity spectrum and investigate strain selectivity of GlcEnt–Amp/Amx, we performed mixed-species assays to determine whether these conjugates will selectively kill pathogenic *E. coli* that express IroN cultured in the presence of other organisms. These assays confirmed that GlcEnt–Amp/Amx **7–10** selectively kill pathogenic *E. coli* that express IroN in the presence of *E. coli* strains that do not express this receptor. Treatment of co-cultures of pathogenic *E. coli* (CFT073 or UTI89, transformed with the chloramphenicol resistance plasmid pSG398) and non-pathogenic *E. coli* K-12 (transformed with the kanamycin resistance plasmid pET29a) with 100 nM Ent–Amp/Amx **5** and **6** results in complete killing of both strains (Fig. 5a–d and S16a–d†). In contrast, treatment of the co-cultures with 100 nM GlcEnt–Amp/Amx **7–10** affords killing of the uropathogenic *E. coli* concomitant with *E. coli* K-12 survival (Fig. 5a–d and S16a–d†). Taken together, these results demonstrate that GlcEnt–β-lactam conjugates **7–10** provide strain-specific targeting of the antibacterial cargo to virulent *E. coli* that express IroN.

(Glc)Ent–Amp/Amx **5–10** also target pathogenic *E. coli* in the presence of commensal microbes. Lactobacilli are Gram-positive commensal bacteria of the human gastrointestinal tract, and are also found in the urinary and genital tracts.^61^ Some Lactobacilli reduce recurrent urinary tract infections in women.^62^ Lactobacilli have little-to-no minimal metabolic iron requirement, and do not employ enterobactin or salmochelins for iron acquisition.^63,64^ *Lactobacillus rhamnosus* GG (ATCC 53103), a human commensal that is considered to be a probiotic, is susceptible to β-lactam antibiotics, and we obtained a MIC value of 10 μM for Amp/Amx against this strain (1 : 1 MRS/MHB medium, ±DP) (Fig. S17†). In contrast, 10 μM (Glc)Ent–Amp/Amx **5–10** have negligible effect on *L. rhamnosus* GG growth (Fig. S17†). Treatment of *E. coli* CFT073 and *L. rhamnosus* GG co-cultures with (Glc)Ent–Amp/Amx **5–10** affords selective killing of *E. coli* CFT073 (Fig. 5e and f, S16e and f†).

We previously reported that modification of Amp/Amx with Ent attenuated the activity of the β-lactam against *Staphylococcus aureus* ATCC 25923.^44^ In the current work we obtained a similar result with GlcEnt–Amp/Amx, and found that the salmochelin modification lowers the antibacterial activity of Amp/Amx against *S. aureus* by 10-fold (Fig. S18†). Moreover, treatment of *E. coli* CFT073 and *S. aureus* co-cultures with DGE–Amp/Amx **9**,**10** affords selective killing of *E. coli* CFT073 (Fig. S19a and b, S20a and b†). Selective killing of *E. coli* CFT073 co-cultured with *Acinetobacter baumannii* ATCC 17961 also occurred (Fig. S19c and d, S20c and d, S21†). Substitution of *E. coli* CFT073 with UTI89 in these assays afforded similar selectivity trends (Fig. S22 and S23†). In total, the mixed-species assays provide support for DGE-based targeting of the antibacterial cargo to IroN-expressing strains.

### GlcEnt–Amp/Amx kill *E. coli* in the presence of lipocalin-2

**Fig. 6:**
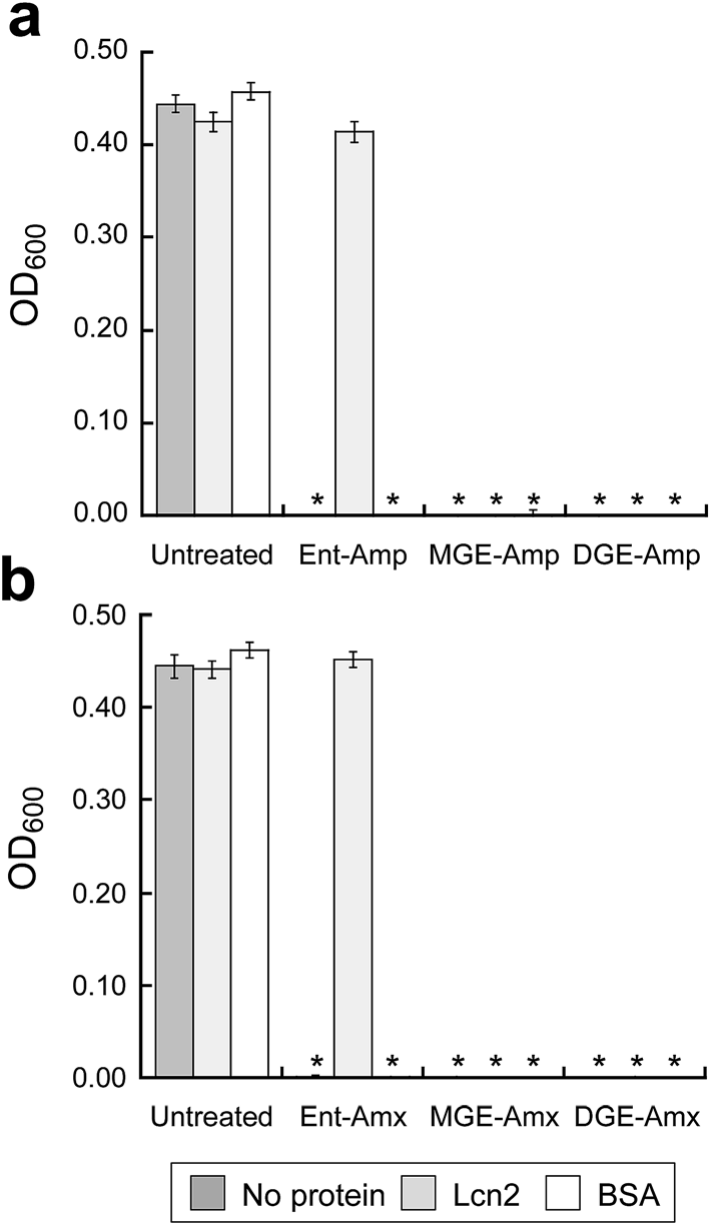
Antibacterial activity of (Glc)Ent–Amp/Amx against *E. coli* CFT073 in the presence of Lcn2 or bovine serum albumin (BSA). *E. coli* CFT073 was treated with (a) 100 nM (Glc)Ent–Amp **5**/**7**/**9** or (b) 100 nM (Glc)Ent–Amx **6**/**8**/**10** in the absence (control) and presence of 1 μM Lcn2 or 1 μM BSA. The assays were conducted in modified M9 medium (*t* = 24 h, *T* = 37 °C) (mean ± standard deviation, *n* = 3). An asterisk indicates OD_600_ < 0.01. The data for the conjugates preloaded with Fe(iii) are presented in Fig. S24.†

To ascertain whether GlcEnt–Amp/Amx **7–10** overcome Lcn2 sequestration, in analogy to Lcn2 evasion by the salmochelins,^14,19^ we conducted antibacterial assays with *E. coli* CFT073 in the absence or presence of Lcn2 or bovine serum albumin (BSA, control). These assays were conducted in modified M9 medium,^65^ and 100 nM (Glc)Ent–Amp/Amx **5–10** provide complete growth inhibition of *E. coli* CFT073 in this medium (Fig. 6). A 10-fold excess of Lcn2 attenuates the antibacterial activity of Ent–Amp/Amx **5** and **6**, in agreement with prior work.^44^ In contrast, Lcn2 has negligible effect on the antimicrobial activity of GlcEnt–Amp/Amx **7–10** against *E. coli* CFT073 (Fig. 6 and S24†).

### GlcEnt–Amp/Amx exhibit low cytotoxicity to human T84 cells

The cytotoxicity of apo and iron-bound GlcEnt–Amp/Amx **7–10** (≤10 μM) against human T84 colon epithelial cells was evaluated by the MTT assay. In all cases, the cell viability was ≥80% of that of the untreated control, indicating that the conjugates exhibit negligible cytotoxicity to T84 cells over a 24 h period (Fig. S25†).

## Conclusions

In this work, inspired by the siderophore recognition strategies utilised by *E. coli* for iron acquisition in the host, we report a siderophore-based approach for antibiotic delivery that targets strains that express IroN, a siderophore receptor that contributes to virulence. First, we establish that the tailoring enzymes IroB and MceC can C-glucosylate monofunctionalized Ent and therefore be employed in chemoenzymatic synthesis to afford functionalized salmochelins. Next, we demonstrate that GlcEnt–β-lactam conjugates are recognised by siderophore transport machinery, target IroN, provide ≥100-fold enhanced antibacterial activity against uropathogenic *E. coli* relative to the parent β-lactams, afford killing of virulent *E. coli* in the presence of non-pathogenic *E. coli* and other commensal strains, and overcome the enterobactin–sequestering host-defense protein Lcn2. Our results establish that conjugation of a broad-spectrum antibiotic to a siderophore tunes the activity profile of the parent antibiotic. With the appropriate choice of siderophore, the antibacterial activity spectrum can be modulated to afford species- and strain-specific targeting. In broad terms, targeting pathogens is important for pharmaceutical development, which will ultimately afford treatment options that minimally perturb the commensal microbiota.^66,67^

IroN was first discovered in *Salmonella* ^18^ and subsequently identified in other Enterobacteriaceae. Our current work focuses on antibiotic delivery to uropathogenic *E. coli* that harbour the *iroA* gene cluster, and we expect that this strategy will be applicable to other pathogens that employ salmochelins for iron acquisition. At present, 121 completely sequenced *E. coli* genomes are available, which include 46 human pathogens. A BLAST search using *iroN* from *E. coli* CFT073 afforded hits with ≥99% sequence identity for three uropathogenic *E. coli* (UTI89, 536, and 83792), adherent invasive *E. coli* UM146, the meningitis isolate *E. coli* IHE3034, and a carbapenemase-producing isolate *E. coli* ECONIH1 (Table S4†). The probiotic *E. coli* Nissle 1917 and the laboratory reference strain for antimicrobial testing *E. coli* ATCC 25922 were the only other *E. coli* revealed as hits. Studies of the distribution of siderophore biosynthetic machinery in *E. coli* isolated from feces of healthy mammals indicate that ≈20% of the commensal isolates produce salmochelins.^68^ This observation suggests that one potential limitation of GlcEnt-based antibiotic delivery is that a fraction of commensal *E. coli* harbour the *iroA* cluster are susceptible and, conversely, that some pathogenic *E. coli* do not. Regarding the former possibility, the healthy gut is considered to be a reservoir for *E. coli* that cause infections of the urinary tract,^58,69–71^ and the ability to target such pathobionts using siderophores may be advantageous in certain cases. In addition to *Salmonella* and *E. coli*, BLAST revealed that the genomes of the human pathogens *Shigella dysenteriae* 1617 and Sd197, *Enterobacter cloacae*, *Klebsiella pneumoniae*, and *Enterobacter aerogenes* encode *iroN* (Table S4†). Thus, it will be informative to determine whether DGE also provides targeted antibiotic delivery to these problematic strains.

Our current investigations also provide fundamental insights into siderophore recognition and transport. Prior studies of siderophore uptake in *Salmonella* revealed that both FepA and IroN recognise and transport Ent.^72^ Our competition assays employing uropathogenic *E. coli* are in agreement with this observation, and indicate that both receptors deliver Ent–Amp/Amx **5** and **6** into *E. coli*. Moreover, our competition data suggest that MGE **2** and MGE–Amp/Amx **7** and **8** are recognised and transported by FepA as well as IroN of *E. coli*. In contrast, DGE **3** only competes with GlcEnt–Amp/Amx **7–10** and most effectively blocks the activity of DGE–Amp/Amx **9** and **10**. These observations support exclusive transport of DGE–Amp/Amx **9** and **10** through IroN. Indeed, prior studies demonstrated that IroN is required for transporting salmochelin extracts isolated from several *S. enterica* strains,^16^ and *in vitro* activity assays reveal that IroB accumulates DGE **3**.^51^

We previously reported that *E. coli* CFT073 exhibits greater sensitivity to Ent–Amp/Amx **5** and **6** than *E. coli* UTI89,^44^ and we observe the same trend with GlcEnt–Amp/Amx **7–10**. The physiological origins of this observation remain unclear. One possible explanation may be differences in the siderophore biosynthetic and uptake machineries employed by these two uropathogens. *E. coli* CFT073 expresses a third catecholate siderophore receptor, IhA,^73^ whereas *E. coli* UTI89 biosynthesizes yersiniabactin, a siderophore mainly used by *Yersinia* spp.^74^ Alternatively, as-yet unidentified factors may account for these trends, and further studies are warranted to understand these observations.

In closing, this investigation establishes that siderophores and the siderophore uptake machinery employed by virulent bacteria provide a powerful approach for targeting pathogenesis in the context of antibacterial drug discovery. Narrow-spectrum and species-specific antibiotics are needed for treating infections where the causative agent is known and, when coupled with rapid diagnostics, will ultimately reduce the onset of secondary infections and evolution of antibiotic resistance.^2,5,7^ The current study focuses on targeting broad-spectrum β-lactam antibiotics to pathogenic *E. coli* on the basis of iron acquisition machinery that is employed by these pathogens during colonisation in the host. We establish that native salmochelins can be used as scaffolds for “Trojan horse” antibiotic delivery to hijack the iron acquisition machinery that contributes to pathogenicity. It will be important to ascertain whether this salmochelin–inspired strategy is applicable to other Gram-negatives, such as *Salmonella* and *K. pneumoniae*, which cause human disease and utilise salmochelins for iron acquisition. Leveraging this strategy to target other antibacterial cargos and thereby modulate activity and mitigate off-target effects is another important avenue of future chemical and biological investigation.

## Experimental section

### Synthetic reagents

Anhydrous dimethyl sulfoxide (DMSO) was purchased from Sigma-Aldrich and used as received. All other chemicals and solvents were purchased from Sigma-Aldrich or Alfa Aesar in the highest available purity and used as received. The syntheses of Ent **1**,^75^ MGE **2**,^52^ DGE **3**,^51^ Ent–Amp **5**,^44^ Ent–Amx **6**,^44^ Ent-PEG_3_-N_3_ **11**,^44^ Amp–alkyne **14**,^44^ and Amx–alkyne **15** ^44^ are reported elsewhere.

### Instrumentation

Analytical and semi-preparative high-performance liquid chromatography (HPLC) were performed using an Agilent 1200 series HPLC system outfitted with a Clipeus reverse-phase C18 column (5 μm pore size, 4.6 × 250 mm; Higgins Analytical, Inc.) at a flow rate of 1 mL min^–1^ and an Agilent Zorbax reverse-phase C18 column (5 μm pore size, 9.4 × 250 mm) at a flow rate of 4 mL min^–1^, respectively. The multi-wavelength detector was set to read the absorbance at 220, 280, and 316 (catecholate absorption) nm. HPLC-grade acetonitrile (MeCN) and trifluoroacetic acid (TFA) were purchased from EMD and Alfa Aesar, respectively. For HPLC analyses, solvent A was 0.1% TFA/H_2_O and solvent B was 0.1% TFA/MeCN, unless stated otherwise. The HPLC solvents were prepared with HPLC-grade MeCN and TFA, and Milli-Q water (18.2 mΩ cm), and filtered through a 0.2 μm filter before use. For analytical HPLC to evaluate conjugate purity, the entire portion of each HPLC-purified compound was dissolved in a mixture of 1 : 1 MeCN/H_2_O and an aliquot was taken for HPLC analysis. The remaining solution was subsequently lyophilized.

High-resolution mass spectrometry was performed by using an Agilent LC-MS system comprised of an Agilent 1260 series LC system outfitted with an Agilent Poroshell 120 EC-C18 column (2.7 μm pore size) and an Agilent 6230 TOF system housing an Agilent Jetstream ESI source. For all LC-MS analyses, solvent A was 0.1% formic acid/H_2_O and solvent B was 0.1% formic acid/MeCN (LC-MS grade, Sigma-Aldrich). The samples were analysed using a solvent gradient of 5–95% B over 10 min with a flow rate of 0.4 mL min^–1^. The MS profiles were analysed and deconvoluted by using Agilent Technologies Quantitative Analysis 2009 software version B.03.02.

Optical absorption spectra were recorded on a Beckman Coulter DU800 spectrophotometer (1 cm quartz cuvettes, Starna). A BioTek Synergy HT plate reader was used to record absorbance at 600 nm (OD_600_) for antimicrobial activity assays and absorbance at 550 nm for cytotoxicity assays.

### Enzymatic activity assays for IroB and MceC

The enzymes MceC and IroB were overexpressed as N-terminal His_6_-fusion proteins in *E. coli* BL21(DE3) and purified as reported.^51,52^ To a 405 μL solution containing Ent-PEG_3_-N_3_ **11** (100 μM), uridine diphosphoglucose (UDP-Glc, 3 mM), and MgCl_2_ (5 mM) prepared in 75 mM Tris–HCl buffer, pH 8.0, MceC (10 μM, 45 μL) or IroB (10 μM, 45 μL) was added to afford a final enzyme concentration of 1 μM. The reaction was incubated at room temperature and an aliquot (100 μL) was quenched by adding 10 μL of 6% TFA (aq) after 0, 15, 30, 60 min. The quenched reaction aliquots were immediately vortexed, centrifuged (13 000 rpm × 10 min, 4 °C), and analysed by HPLC (0–100% B over 30 min, 1 mL min^–1^). The results are shown in Fig. S1 and S2.†

### Synthesis of MGE/DGE-PEG_3_-N_3_ (**12** and **13**)

A 6.3 mL solution containing Ent-PEG_3_-N_3_ **11** (500 μM), uridine diphosphoglucose (UDP-Glc, 3 mM), and MgCl_2_ (5 mM) was prepared in 75 mM Tris–HCl buffer, pH 8.0 and divided into seven 900 μL aliquots. MceC (50 μM, 100 μL) or IroB (50 μM, 100 μL) was added to each aliquot to afford a final enzyme concentration of 5 μM. The 1 mL reactions were incubated at room temperature and quenched by addition of 100 μL of 6% TFA (aq) after 15 min (MceC reaction) or 2 h (IroB reaction). The quenched reaction aliquots were immediately vortexed, combined, and lyophilized to dryness. The resulting powder was dissolved in 3 mL of 1 : 1 MeCN/water and centrifuged (13 000 rpm × 10 min, 4 °C). MGE-PEG_3_-N_3_ **12** and DGE-PEG_3_-N_3_ **13** were purified from the supernatants of MceC- and IroB-catalysed reactions, respectively, by using semi-preparative HPLC (20–45% B over 8.5 min, 4 mL min^–1^). Both compounds were obtained as white powders (MGE-PEG_3_-N_3_ **12**, 0.66 mg, 41% from MceC-catalyzed reaction; DGE-PEG_3_-N_3_ **13**, 0.85 mg, 45% from IroB-catalysed reaction). HRMS (ESI): MGE-PEG_3_-N_3_ **12**, [M + H]^+^ *m*/*z* calcd. 1076.3215, found 1076.3214; DGE-PEG_3_-N_3_ **13**, [M + H]^+^ *m*/*z* calcd. 1238.3743, found 1238.3744. The analytical HPLC traces of the purified compounds are reported in Fig. S26 and S27.†

### Synthesis of MGE–Amp (**7**)

Amp–alkyne **14** (50 μL of a 50 mM solution in DMSO, 2.5 μmol) and MGE-PEG_3_-N_3_ **12** (73 μL of an 11.3 mM solution in DMSO, 0.825 μmol) were combined and 100 μL of DMSO was added. An aliquot of aqueous CuSO_4_ (50 μL of a 90 mM solution in water, 4.5 μmol) and tris[(1-benzyl-1*H*-1,2,3-triazol-4-yl)methyl]amine (TBTA, 100 μL of a 50 mM solution in DMSO, 5 μmol) were combined to give a blue-green solution, to which sodium ascorbate (NaAsc, 100 μL of a 180 mM solution in water, 18.0 μmol) was added. This solution became light yellow and was immediately added to the alkyne/azide solution. The reaction was gently mixed on a bench-top rotator for 2 h at room temperature and conjugate **7** was purified by semi-preparative HPLC (20% B for 5 min and 20–50% B over 11 min, 4 mL min^–1^; 0.005% TFA was used in solvents A and B because of the acid-sensitive β-lactam moiety). Conjugate **7** was obtained as white powder (0.75 mg, 59%). HRMS (ESI): [M + H]^+^ *m*/*z* calcd, 1519.4730; found, 1519.4639. The analytical HPLC trace of the purified product is reported in Fig. S28.†

### Synthesis of MGE–Amx (**8**)

As described for MGE–Amp with the exception that Amx–alkyne **15** was used instead of Amp–alkyne **14**. Conjugate **8** was purified by semi-preparative HPLC (20% B for 5 min and 20–42% B over 11 min, 4 mL min^–1^) and obtained as white powder (0.49 mg, 31%). HRMS (ESI): [M + H]^+^ *m*/*z* calcd, 1535.4679; found, 1535.4685. The analytical HPLC trace of the purified product is reported in Fig. S29.†

### Synthesis of DGE–Amp (**9**)

As described for MGE–Amp with the exception that DGE-PEG_3_-N_3_ **13** was used instead of MGE-PEG_3_-N_3_ **12**. Conjugate **9** was purified by semi-preparative HPLC (0% B for 5 min and 0–50% B over 13 min, 4 mL min^–1^) and obtained as white powder (0.67 mg, 48%). HRMS (ESI): [M + Na]^+^ *m*/*z* calcd, 1703.5077; found, 1703.5069. The analytical HPLC trace of the purified product is reported in Fig. S30.†

### Synthesis of DGE–Amx (**10**)

As described for MGE–Amp with the exception that Amx–alkyne **15** and DGE-PEG_3_-N_3_ **13** were used instead of Amp–alkyne **14** and MGE-PEG_3_-N_3_ **12**. Conjugate **10** was purified by semi-preparative HPLC (0% B for 5 min and 0–50% B over 13 min, 4 mL min^–1^) and obtained as white powder (0.36 mg, 26%). HRMS (ESI): [M + H]^+^ *m*/*z* calcd, 1697.5207; found, 1697.5235. The analytical HPLC trace of the purified product is reported in Fig. S31.†

### Storage and handling of siderophores and siderophore–antibiotic conjugates

All (Glc)Ent **1–3** and siderophore–antibiotic conjugates **5–10** were stored as DMSO stock solutions at –20 °C. The stock solution concentrations for (Glc)Ent–Amp/Amx **5–10** ranged from 2 to 5 mM. These values were determined by diluting the DMSO stock solution in MeOH and using the reported extinction coefficient for enterobactin in MeOH (316 nm, 9500 M^–1^ cm^–1^).^76^ To minimize multiple freeze–thaw cycles, the resulting solutions were divided into 50 μL aliquots and stored at –20 °C. The β-lactam moieties and enterobactin trilactone are susceptible to hydrolysis, and aliquots were routinely analysed by HPLC to confirm the integrity of the samples.

### General microbiology materials and methods

Information pertaining to all bacterial strains used in this study is listed in Table S1.† Freezer stocks of all *Escherichia coli* strains (*E. coli* K-12, B, H9049, CFT073, and UTI89), *Staphylcoccus aureus* ATCC 25923, and *Acinetobacter baumannii* ATCC 17961 were prepared from single colonies in 25% glycerol/Luria Broth (LB) medium. Freezer stocks of *Lactobacillus rhamnosus* GG ATCC 53103 were prepared from single colonies in 25% glycerol/de Man, Rogosa, and Sharpe (MRS) medium.

LB, MRS, 5× M9 minimal medium and agar were purchased from BD. Mueller Hinton Broth (MHB) was purchased from Fluka. Recombinant human Lcn2 was purchased from R&D System (Minneapolis, MN). The iron chelator 2,2′-dipyridyl (DP) was purchased from Sigma-Aldrich. All growth medium and Milli-Q water used for bacterial cultures or for preparing solutions of the enterobactin–antibiotic conjugates were sterilised by using an autoclave. A DP stock solution (200 mM) was prepared in DMSO and used in the bacteria growth assays requiring iron-depleted conditions. Working dilutions of the siderophore and siderophore–antibiotic conjugate stock solutions were prepared in 10% DMSO/H_2_O. For all assays, the final cultures contained 1% v/v DMSO. Sterile polypropylene culture tubes and sterile polystyrene 96-well plates used for culturing were purchased from VWR and Corning Incorporated, respectively. The optical density at 600 nm (OD_600_) was recorded on a Beckman Coulter DU800 spectrophotometer or by using a BioTek Synergy HT plate reader.

### Growth studies of *E. coli* in the presence of DP

Overnight cultures of *E. coli* were prepared by inoculating 5 mL of Luria Broth (LB) medium with bacterial freezer stocks. The cultures were incubated at 37 °C in a tabletop incubator with shaking at 150 rpm for 16–18 h. The overnight culture was diluted 1 : 100 into 5 mL of fresh LB medium containing DP (200 μM) and incubated at 37 °C with shaking at 150 rpm until OD_600_ reached 0.6. The cultures were subsequently diluted to an OD_600_ value of 0.001 using 50% MHB medium (11.5 g L^–1^). A 90 μL aliquot of the diluted culture was combined with a 10 μL aliquot of a 10× solution of DP (0, 0.25, 0.5, 1, 2, 4, and 8 mM) in a 96-well plate, which was wrapped in Parafilm and incubated at 30 °C with shaking at 150 rpm. Bacterial growth was determined at *t* = 0, 2, 4, 6, 8, 10, and 20 h by measuring the OD_600_ using a BioTek Synergy HT plate reader. Each well condition was prepared in duplicate and at least three independent replicates were conducted on different days and using two different DP stock solutions. The resulting mean OD_600_ values are reported and the error bars represent the standard deviation.

### General procedure for antimicrobial activity assays

Overnight cultures of *E. coli*, *S. aureus*, and *A. baumannii* were prepared by inoculating 5 mL of Luria Broth (LB) medium with bacterial freezer stocks. The cultures were incubated at 37 °C in a tabletop incubator with shaking at 150 rpm for 16–18 h. The overnight culture was diluted 1 : 100 into 5 mL of fresh LB medium containing DP (200 μM) and incubated at 37 °C with shaking at 150 rpm until OD_600_ reached 0.6. The cultures were subsequently diluted to an OD_600_ value of 0.001 using 50% MHB medium (11.5 g L^–1^) with or without DP (200 μM). A 90 μL aliquot of the diluted culture was combined with a 10 μL aliquot of a 10× solution of Amp/Amx or (Glc)Ent–Amp/Amx **5–10** in a 96-well plate, which was wrapped in Parafilm and incubated at 30 °C with shaking at 150 rpm for 19 h. Bacterial growth was determined by measuring the OD_600_ using a BioTek Synergy HT plate reader. Each well condition was prepared in duplicate and at least three independent replicates were conducted on different days and using different synthetic batches of each conjugate. The resulting mean OD_600_ values are reported and the error bars represent the standard deviation.

For *L. rhamnosus* GG ATCC 53103, the bacterial culture was grown in MRS medium overnight. The resulting culture was diluted 1 : 50 into 5 mL of fresh MRS medium containing DP (200 μM) and incubated at 37 °C with shaking at 150 rpm until OD_600_ reached 1.0. The culture was subsequently diluted to an OD_600_ value of 0.004 in 1 : 1 MRS/MHB medium with or without DP (200 μM). The antibacterial activity assays were performed as described above for *E. coli*.

### Time-kill kinetic assays

A 5 mL overnight culture of *E. coli* CFT073 or UTI89 grown in LB medium was diluted 1 : 100 into 5 mL of fresh LB medium with 200 μM DP and incubated at 37 °C with shaking at 150 rpm until OD_600_ reached ≈0.3. The culture was centrifuged (3000 rpm × 10 min, rt) and the resulting pellet was resuspended in 50% MHB and centrifuged (3000 rpm × 10 min, rt). The resulting pellet was resuspended in 50% MHB with or without DP (200 μM) and the OD_600_ was adjusted to 0.3. A 90 μL aliquot of the resulting culture was combined with a 10 μL aliquot of a 10× solution of Amp/Amx or (Glc)Ent–Amp/Amx **5–10** in a 96-well plate, which was wrapped in Parafilm and incubated at 37 °C with shaking at 150 rpm. The OD_600_ values were recorded at *t* = 0, 1, 2, and 3 h. In a parallel experiment, a 10 μL aliquot of the culture was taken at *t* = 0, 1, 2, and 3 h and serially diluted by using sterile phosphate-buffered saline (PBS) and plated on LB agar to obtain colony forming units (CFU mL^–1^). Each well condition was repeated at least three times independently on different days. The resulting mean OD_600_ or CFU mL^–1^ is reported and the error bars are the standard deviation.

### Antimicrobial activity assays in the presence of unmodified (Glc)Ent

These assays were performed following the general procedure described above except that varying concentrations of Ent, MGE, or DGE were mixed with Ent–Amp/Amx **5** and **6**, MGE–Amp/Amx **7–8**, or DGE–Amp/Amx **9** and **10**. Ent was synthesized following a literature procedure,^75^ MGE **2** and DGE **3** were prepared from Ent using MceC and IroB as described for MGE-PEG_3_-N_3_ **12** and DGE-PEG_3_-N_3_ **13**. Stock solutions of (Glc)Ent **1–3** were prepared in DMSO and stored at –20 °C.

### Mixed-*E. coli* assays

The pET29a plasmid (kanamycin resistance) was transformed into *E. coli* K-12, and the pHSG398 plasmid (chloramphenicol resistance) was transformed into *E. coli* CFT073 and UTI89, by electroporation. Overnight cultures of the bacterial strains were prepared by inoculating 5 mL of LB medium containing the appropriate antibiotic (kanamycin, 50 μg mL^–1^; chloramphenicol, 34 μg mL^–1^) with bacterial freezer stocks, and the cultures were incubated at 37 °C in a tabletop incubator shaker set at 150 rpm for 16–18 h. Each overnight culture was diluted 1 : 100 into 5 mL of fresh LB medium containing 200 μM DP, but no antibiotics, and incubated at 37 °C with shaking at 150 rpm until OD_600_ reached 0.6. The cultures were diluted to an OD_600_ value of 0.001 in 50% MHB separately or in a 1 : 1 mixture (10^6^ CFU mL^–1^ for each strain), with or without 200 μM DP. No antibiotic marker was included in these cultures. Aliquots of these cultures were serially diluted by using sterile PBS and plated on LB agar plates with or without corresponding antibiotic to confirm the CFU of the starter cultures. A 90 μL aliquot of each culture was combined with a 10 μL aliquot of a 1 μM solution of Amp/Amx or (Glc)Ent–Amp/Amx **5–10** in a 96-well plate. The plate was then wrapped in Parafilm and incubated at 30 °C with shaking at 150 rpm for 19 h. Bacterial growth was evaluated by measuring OD_600_ as well as counting colonies formed on LB agar with or without kanamycin/chloramphenicol after serial dilution with sterile PBS. Each well condition was repeated at least three times independently on different days. The resulting mean OD_600_ and CFU mL^–1^ values are reported and the error bars are the standard deviation.

### Mixed-species assays

These assays were performed following the mixed-*E. coli* assay procedure except that *E. coli* CFT073 and *L. rhamnosus* GG ATCC 53103 were used. A 5 mL culture of *E. coli* CFT073 or *L. rhamnosus* GG was grown for 16–18 h in LB or MRS medium, respectively. The overnight culture was diluted 1 : 100 (*E. coli*) or 1 : 50 (*L. rhamnosus* GG) into 5 mL of fresh LB or MRS medium with 200 μM DP and incubated at 37 °C with shaking at 150 rpm until OD_600_ reached 0.6 (*E. coli*) or 1.0 (*L. rhamnosus* GG). The cultures were diluted to an OD_600_ value of 0.001 (*E. coli*) or 0.004 (*L. rhamnosus* GG) in 1 : 1 MRS/MHB containing 200 μM DP separately or in a 1 : 1 mixture (10^6^ CFU mL^–1^ for each strain). Aliquots of these cultures were serially diluted by using sterile PBS and plated on LB and MRS agar plates to confirm the CFU of the starter culture. A 90 μL aliquot of each culture was combined with a 10 μL aliquot of a 10 μM solution of Amp/Amx or (Glc)Ent–Amp/Amx **5–10** in a 96-well plate, which was wrapped in Parafilm and incubated at 30 °C with shaking at 150 rpm for 19 h. Bacterial growth was assayed by both measuring OD_600_ and counting colonies formed on LB and MRS agar plates after serial dilution with sterile PBS. Each well condition was repeated at least three times independently on different days. The resulting mean OD_600_ and CFU mL^–1^ values are reported and the error bars are the standard deviation. Comment: *E. coli* CFT073 forms colonies more quickly than *L. rhamnosus* GG on LB agar plates, whereas *L. rhamnosus* GG colonies appear more quickly than those of *E. coli* CFT073 on MRS agar plates, and these behaviours allow for each strain to be monitored independently over a 24 h period.

The assays were also performed by co-culturing *E. coli* CFT073 or UTI89 with *S. aureus* ATCC 25923 or *A. baumannii* ATCC 17961. A 5 mL culture of each individual bacterial strain was grown for 16–18 h in LB. The overnight culture was diluted 1 : 100 into 5 mL of fresh LB with 200 μM DP and incubated at 37 °C with shaking at 150 rpm until OD_600_ reached 0.6. The cultures were diluted to an OD_600_ value of 0.001 in 50% MHB containing 200 μM DP separately or in a 1 : 1 mixture (10^6^ CFU mL^–1^ for each strain). A 90 μL aliquot of each culture was combined with a 10 μL aliquot of a 10 μM solution of Amp/Amx or (Glc)Ent–Amp/Amx **5–10** in a 96-well plate, which was wrapped in Parafilm and incubated at 30 °C with shaking at 150 rpm for 19 h. Bacterial growth was assayed by both measuring OD_600_ and counting colonies formed on HardyCHROM UTI plates after serial dilution with sterile PBS. Plating *E. coli* strains on these plates results in pink colonies, whereas *S. aureus* and *A. baumannii* provide white colonies. Each well condition was repeated at least three times independently on different days. The resulting mean OD_600_ and CFU mL^–1^ values are reported and the error bars are the standard deviation.

### Antimicrobial activity assays in the presence of lipocalin **2**

Cultures of *E. coli* CFT073 were grown in modified M9 minimal medium^65^ (Na_2_HPO_4_ 6.8 g L^–1^, KH_2_PO_4_ 3 g L^–1^, NaCl 0.5 g L^–1^, NH_4_Cl 1 g L^–1^, 0.4% glucose, 2 mM MgSO_4_, 0.1 mM CaCl_2_, 0.2% casein amino acids, and 16.5 μg mL^–1^ of thiamine) for 16–18 h. The overnight culture grew to saturation and was diluted 1 : 100 into 5 mL of fresh modified M9 minimal medium and incubated at 37 °C with shaking at 150 rpm until OD_600_ reached 0.6. The OD_600_ of the culture was adjusted to 0.001, and the culture was further diluted 1 : 100 with the M9 medium in two steps (1 : 10 × 1 : 10). The corresponding CFU was determined to be ≈10^4^ CFU mL^–1^ by plating on LB agar plates. Lipocalin 2 (Lcn2, R&D Systems) was diluted into PBS, pH 7.4 to a concentration of 20 μM and frozen at –20 °C until use. Bovine serum albumin (BSA, Sigma-Aldrich) was prepared in PBS, pH 7.4 to achieve a concentration of 20 μM. A 90 μL aliquot of the diluted culture was combined with a 5 μL aliquot of a 20× solution of (Glc)Ent–Amp/Amx **5–10** and a 5 μL aliquot of Lcn2 or BSA in a 96-well plate, which was wrapped in Parafilm and incubated at 37 °C with shaking at 150 rpm for 24 h. Bacterial growth was determined by OD_600_. Each well condition was repeated at least three times independently on different days. The resulting mean OD_600_ is reported and the error bars are the standard deviation.

### Cytotoxicity assays

The human colon epithelial T84 cell line was purchased from ATCC and cultured in 1 : 1 DMEM/F12 medium with 10% fetal bovine serum, and 1% penicillin and streptomycin (v/v, ATCC). The cells were grown to approximately 95% confluency and treated with 3 mL of trypsin-EDTA (Corning). A 12 mL portion of fresh medium was added to the detached cells, and the T84 cell suspension was centrifuged (600 rpm × 5 min, 37 °C). The supernatant was discarded and the cell pellet was resuspended in 6 mL of the fresh culture medium. The concentration of cells was quantified by using a manual hemocytometer (VWR International) and adjusted to 1 × 10^5^ cells per mL. A 90 μL aliquot of T84 cells were then added to 96-well plates and incubated at 37 °C and 5% CO_2_ for 24 h. Stock solutions (10×) of Amp/Amx or (Glc)Ent–Amp/Amx **5–10** were prepared in sterile-filtered 10% DMSO/H_2_O and 10 μL of each solution was added to the appropriate well. The plate was incubated at 37 °C and 5% CO_2_ for another 24 h. 3-[4,5-Dimethylthiazol-2-yl]-2,5 diphenyl tetrazolium bromide (MTT, Alfa Aesar) was dissolved in sterile PBS and the concentration was adjusted to 5 mg mL^–1^. The resulting yellow solution was filtered through a 0.2 μm filter and a 20 μL aliquot of the resulting MTT solution was added to each well. The plate was incubated at 37 °C and 5% CO_2_ for 4 h and the supernatant was removed from each well. DMSO (100 μL) was added to each well and the absorbance at 550 nm was recorded by using a plate reader. Blank readings were recorded on wells that contained only the medium. The assay was repeated in triplicate on different days, and the mean and standard deviation are reported.

## Competing financial interests

A patent application covering GlcEnt–Amp/Amx has been filed.
