# Supplementary material for: Targeting virulence: salmochelin modification tunes the antibacterial activity spectrum of β-lactams for pathogen-selective killing of Escherichia coli
Source: Chem Sci. 2015 May 22;6(8):4458–71. doi: 10.1039/c5sc00962f (PMC5499518; doi:10.1039/c5sc00962f)
Supplement: Supplementary file 1 [file SC-006-C5SC00962F-s001.pdf]

Electronic Supplementary Material (ESI) for Chemical Science.
This journal is © The Royal Society of Chemistry 2015

Supplementary Information for

Targeting Virulence: Salmochelin Modification Tunes the Antibacterial Activity Spectrum of β-Lactams for Pathogen-Selective Killing of *Escherichia coli*

Phoom Chairatana, Tengfei Zheng, and Elizabeth M. Nolan*

Department of Chemistry, Massachusetts Institute of Technology, Cambridge, MA 02139, USA

*Corresponding author: lnolan@mit.edu

Phone: 617-452-2495

Fax: 617-324-0505

This supplementary information includes:

Supplementary Tables.. S3
Table S1. Bacterial strains employed in this study.............................. S3
Table S2. Iron content of the antimicrobial assay medium.......................... S4
Table S3. Characterization of compounds **7-10,12-13**.............................. S4
Table S4. BLAST search for *iroN* sequence.. S5
Supplementary Figures.. S6
Fig. S1. Analytical HPLC of Ent-PEG$_3$-N$_3$ **11** incubated with MceC.................. S6
Fig. S2. Analytical HPLC of Ent-PEG$_3$-N$_3$ **11** incubated with IroB.................... S6
Fig. S3. Optical absorption spectra of MGE/DGE-Amp/Amx............................. S7
Fig. S4. Growth curves of *E. coli* strains in the presence of DP......................... S8
Fig. S5. Antimicrobial activity assays against *E. coli* CFT073............................ S9
Fig. S6. Antimicrobial activity assays against *E. coli* UTI89.............................. S10
Fig. S7. Antimicrobial activity assays against *E. coli* H9049.............................. S11
Fig. S8. Antimicrobial activity assays against *E. coli* K-12................................. S12
Fig. S9. Antimicrobial activity assays against *E. coli* B...................................... S13
Fig. S10. Antibacterial activity assays with conjugates preloaded with Fe(III)............. S14
Fig. S11-S12. Antimicrobial activity of mixtures containing Amp/Amx and (Glc)Ent.... S15
Fig. S13. Time-kill kinetics against *E. coli* CFT073.. S17
Fig. S14. Time-kill kinetics against *E. coli* UTI89... S18
Fig. S15. Competition assays for FepA and IroN recognition.................................. S19
Fig. S16. *E. coli* CFT073 and K-12 / *L. rhamnosus* GG treated with (Glc)Ent-Amx..... S20
Fig. S17. Antimicrobial activity against *L. rhamnosus* GG...................................... S21
Fig. S18. Antimicrobial activity assays against *S. aureus*...................................... S22
Fig. S19. *E. coli* CFT073 and *S. aureus*/*A. baumannii* treated with (Glc)Ent-Amp...... S23
Fig. S20. *E. coli* CFT073 and *S. aureus*/*A. baumannii* treated with (Glc)Ent-Amx....... S24
Fig. S21. Antimicrobial activity assays against *A. baumannii*.................................. S25
Fig. S22. *E. coli* UTI89 and *S. aureus*/*A. baumannii* treated with (Glc)Ent-Amp......... S26
Fig. S23. *E. coli* UTI89 and *S. aureus*/*A. baumannii* treated with (Glc)Ent-Amx.......... S27
Fig. S24. Antimicrobial activity of conjugates in the presence of Lcn2........................ S28
Fig. S25. Cytotoxicity assays against T84 cells... S28
Fig. S26-S31. Analytical HPLC of purified compounds **7-10,12,13**.............................. S29
Supplementary References.. S32

Table S1. Bacterial strains employed in this study.

Strain	Source	Comments
E. coli CFT073	ATCC	Clinical isolate, uropathogenic
		Salmochelin production
		FepA, IroN expression
E. coli UTI89	Prof. L. Cegelski	Clinical isolate, uropathogenic
	(Stanford University)	Salmochelin production
		FepA, IroN expression
E. coli H9049	Prof. C. T. Walsh	Clinical isolate
		FepA expression
E. coli K-12	ATCC	Common lab strain, BL1
		FepA expression
E. coli B	ATCC	Common lab strain, BL1
		FepA expression
L. rhamnosus GG	ATCC	Clinical isolate, probiotic
		Minimal metabolic iron requirement
S. aureus	ATCC	Clinical isolate, pathogenic
		No FepA or IroN expression
A. baumannii	ATCC	Clinical isolate, pathogenic
		No FepA or IroN expression

Iron Content of the Antimicrobial Assay Medium

The iron content of the antimicrobial assay medium was determined by ICP-OES (University of Illinois Urbana-Champaign, UIUC) for two independently prepared batches. Both values are reported.

Table S2. Iron content of the antimicrobial assay medium.[a]

Medium	Fe concentration (ppm)	Fe concentration (μM)
Luria Broth (LB)[b]	0.339	6.05
	0.342	6.11
50% Mueller Hinton Broth (MHB)[b]	0.265	4.73
	0.173	3.09
Modified M9 minimal medium[c]	0.018	0.323
	0.019	0.341
1:1 MRS/MHB medium[c]	0.624	11.2
	0.647	11.6

[a] The samples were stored in polypropylene tubes, and sent to UIUC for ICP-OES analysis. The samples were stored at room temperature and shipped at ambient temperature. [b] Data previously reported.[1] [c] Growth media was prepared as described in the main text and autoclaved.

Table S3. Characterization of compounds **7-10,12,13**.

Compound	Name	HPLC retention time (min)[a]	Observed *m/z*[b]	Calculated *m/z*[b]
**7**	MGE-Amp	22.8	1519.4639	1519.4730
**8**	MGE-Amx	21.6	1535.4685	1535.4679
**9**	DGE-Amp	21.3	1703.5069[c]	1703.5077[c]
**10**	DGE-Amx	19.9	1697.5235	1697.5207
**12**	MGE-PEG$_3$-N$_3$	22.0	1076.3214	1076.3215
**13**	DGE-PEG$_3$-N$_3$	20.0	1238.3744	1238.3743

[a] HPLC gradient used for all compounds is 0% B for 5 min followed by 0-100% B over 30 min, 1 mL/min. [b] All *m/z* values correspond to $[M+H]^+$ unless specified otherwise. [c] The *m/z* value corresponds to $[M+Na]^+$.

BLAST Search for *iroN* Sequence

The *iroN* sequence (2,178 bp) from *E. coli* CFT073 was used to search the sequences deposited in the nucleotide collection of GenBank (as of May 14, 2015) using the BLASTN 2.2.30+ program (http://blast.ncbi.nlm.nih.gov/Blast.cgi).[2] The results (≤78% sequence identity) are summarized below.

Table S4. Results from BLAST search using *iroN* of *E. coli* CFT073.

Species	% Sequence Identity	Comments	Ref.
E. coli ATCC 25922	100	Laboratory reference strain for antimicrobial testing	3
E. coli Nissle 1917	100	Probiotic	4
E. coli CFT073	100	Uropathogenic	5
E. coli 83972	99	Uropathogenic, asymptomatic bacteriuria	6
E. coli 536	99	Uropathogenic	7
E. coli IHE3034	99	Meningitis	8
S. dysenteriae 1617	99	Shigellosis	9
S. dysenteriae Sd197	99	Shigellosis	10
E. coli UM146	99	Adherent invasive, Crohn's disease	11
E. coli UTI89	99	Uropathogenic	12
E. coli ECONIH1	99	Carbapenemase-producing isolate	13
E. cloacae ECNIH2	91	Carbapenemase-producing isolate	13
K. pneumoniae ATCC 43816 KPPR1	91	Uropathogenic, rifampin-resistant isolate	14
K. pneumoniae NTUH-K2044	91	Liver abscess and meningitis	15
E. aerogenes EA1509E	83	Pandrug-resistant isolate	16
E. aerogenes KCTC 2190	83	Laboratory reference strain for antimicrobial testing	17
Salmonella enterica	78	Salmonellosis	18

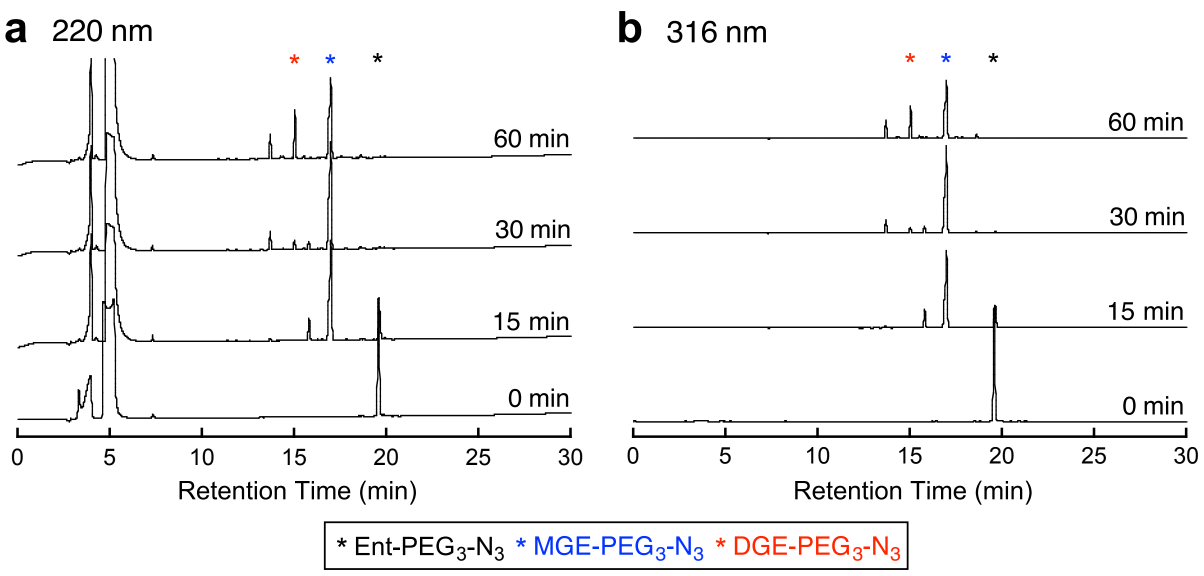

Fig. S1. Analytical HPLC traces of 100 μL of 100 μM Ent-PEG$_3$-N$_3$ **11** incubated with 1 μM MceC in 100 mM Tris-HCl, pH 8.0, 5 mM $MgCl_2$, and 3 mM UDP-Glc for the indicated time. The samples were quenched with 10 μL of 6% TFA. Method: 0-100% B over 30 min, 1 mL/min. (a) Absorbance monitored at 220 nm. (b) Absorbance monitored at 316 nm.

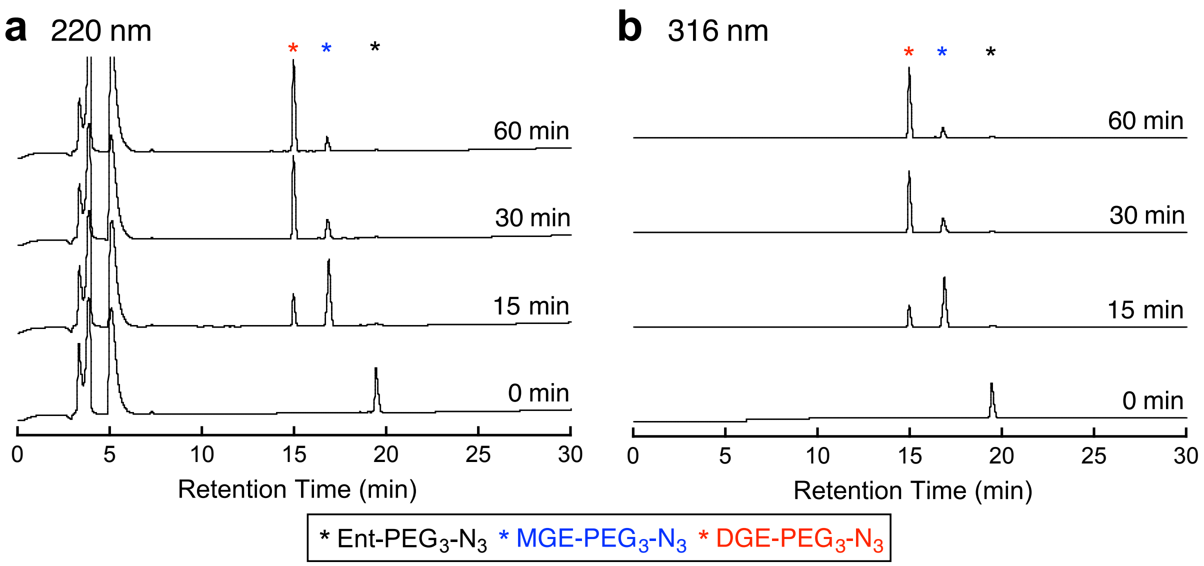

Fig. S2. Analytical HPLC traces of 100 μL of 100 μM Ent-PEG$_3$-N$_3$ **11** incubated with 1 μM IroB in 100 mM Tris-HCl, pH 8.0, 5 mM $MgCl_2$, and 3 mM UDP-Glc for the indicated time. The samples were quenched with 10 μL of 6% TFA. Method: 0-100% B over 30 min, 1 mL/min. (a) Absorbance monitored at 220 nm. (b) Absorbance monitored at 316 nm.

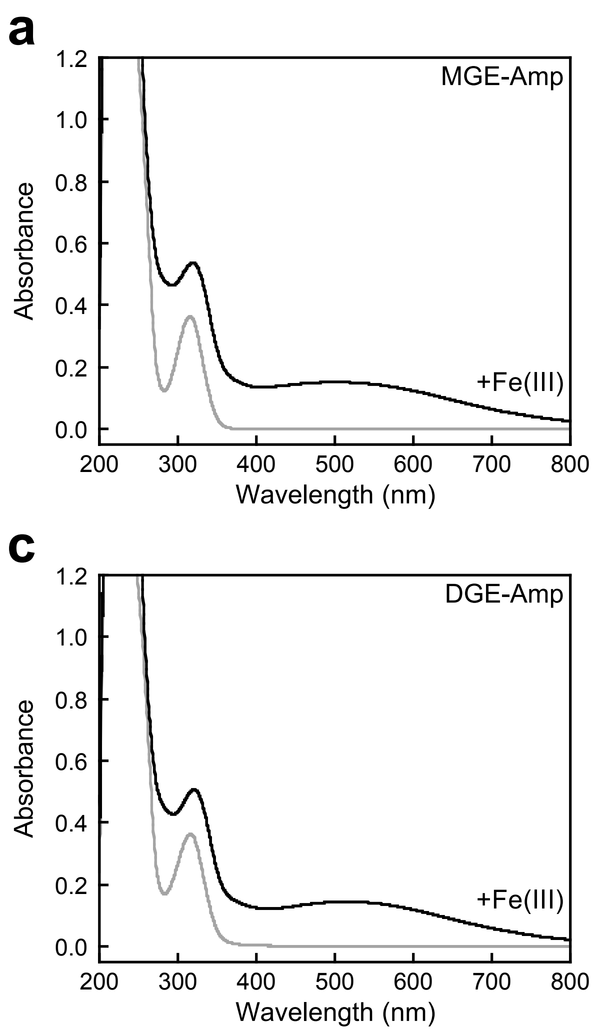

Fig. S3. Optical absorption spectra of 40 μM GlcEnt-Amp/Amx in the absence (grey) and presence (black) of 0.95 equivalents of Fe(III) (MeOH, rt). (a) MGE-Amp **7**. (b) MGE-Amx **8**. (c) DGE-Amp **9**. (d) DGE-Amx **10**.

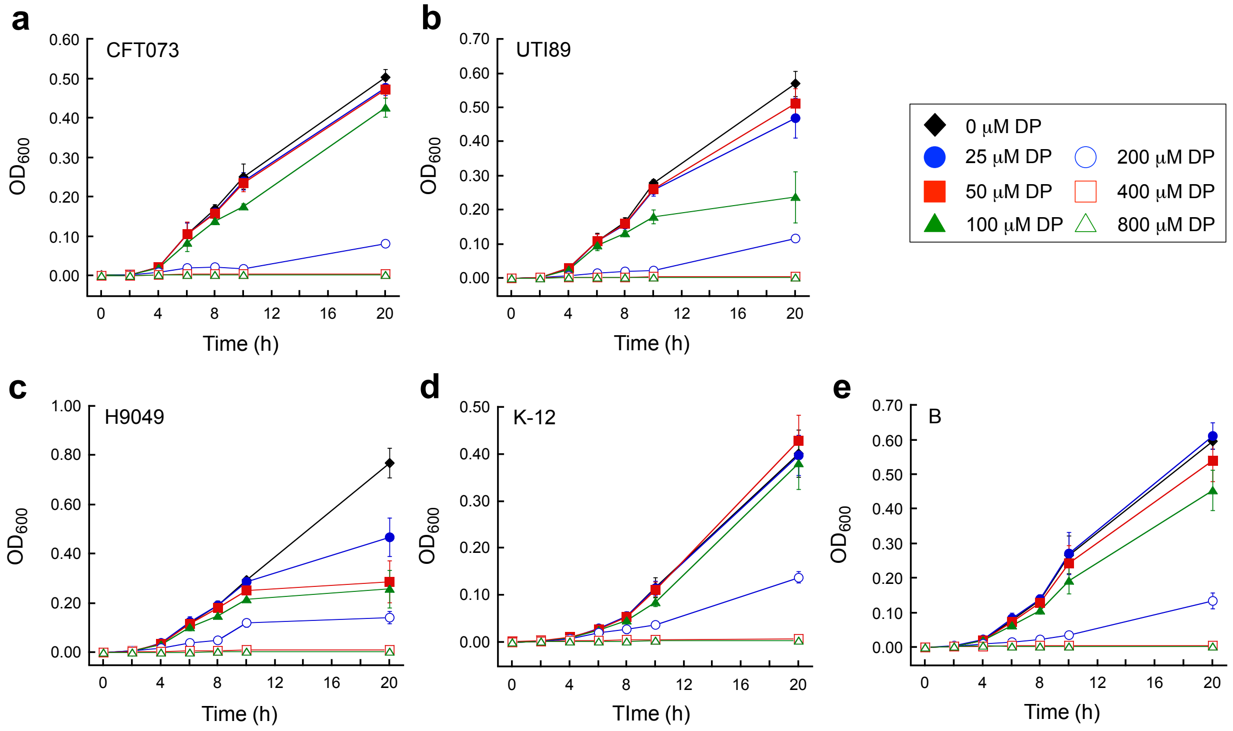

Fig. S4. Growth curves of *E. coli* strains employed in this work in 50% MHB and in the presence of varying concentrations of DP (T = 30 °C) (mean ± standard deviation, n = 3).

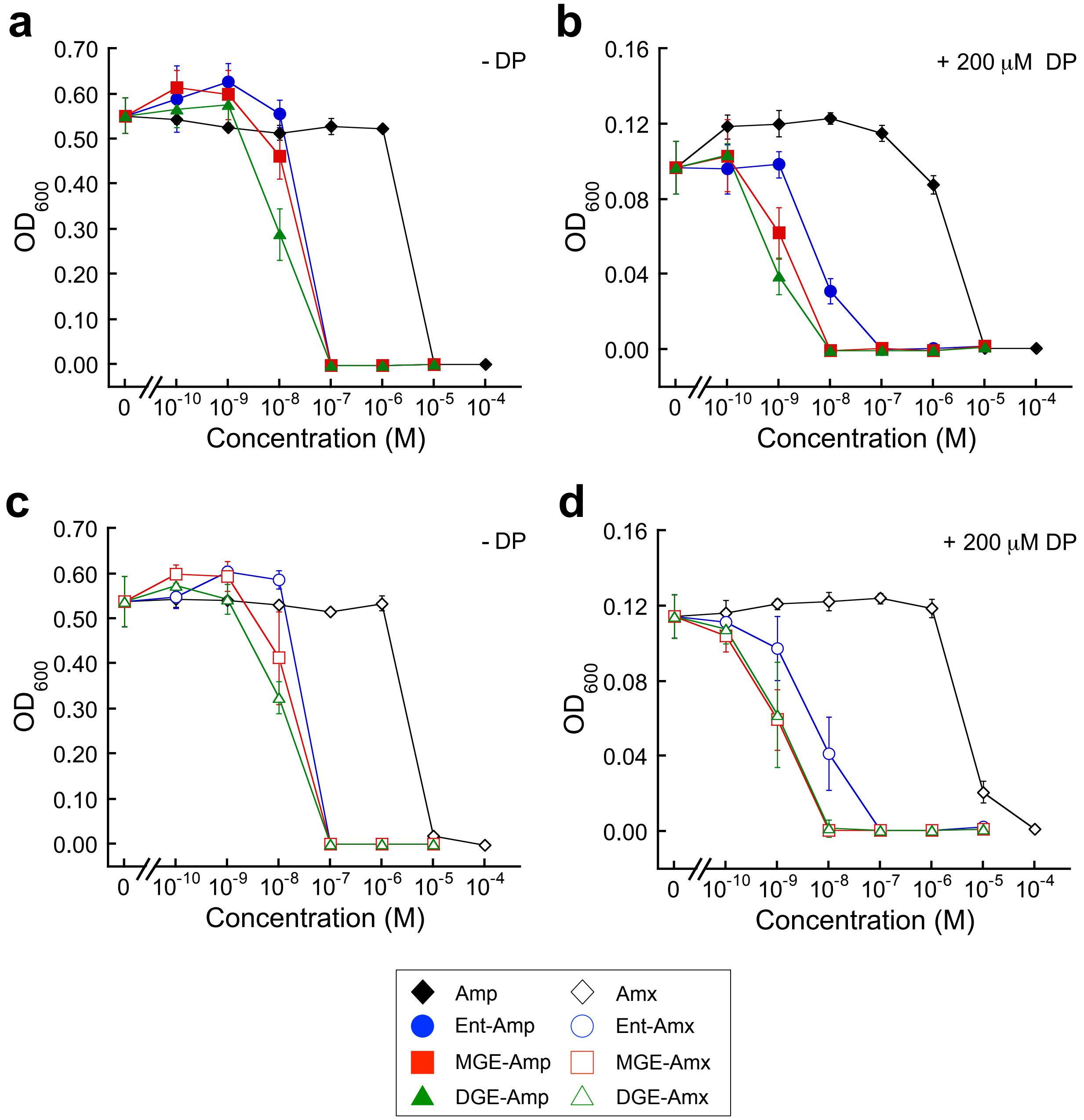

Fig. S5. Antibacterial activity of (Glc)Ent-Amp/Amx **5-10** against *E. coli* CFT073 in 50% MHB medium in the absence and presence of 200 μM DP (t = 19 h, T = 30 °C) (mean ± standard deviation, n = 3). Panel b corresponds to Fig. 2a and is included for direct comparison.

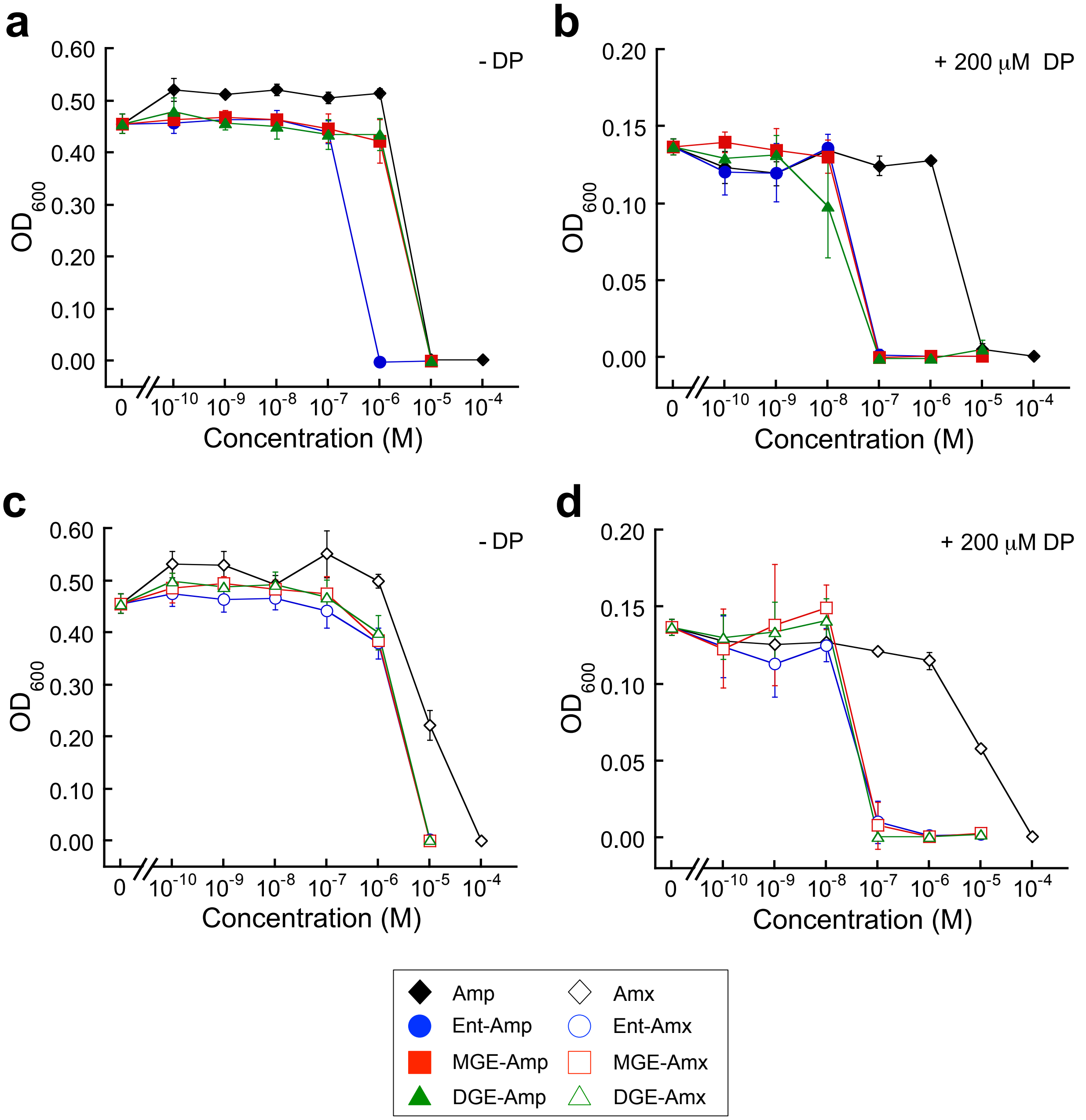

Fig. S6. Antibacterial activity of (Glc)Ent-Amp/Amx **5-10** against *E. coli* UTI89 in 50% MHB medium in the absence and presence of 200 μM DP (t = 19 h, T = 30 °C) (mean ± standard deviation, n = 3). Panel b corresponds to Fig. 2b and is included for direct comparison.

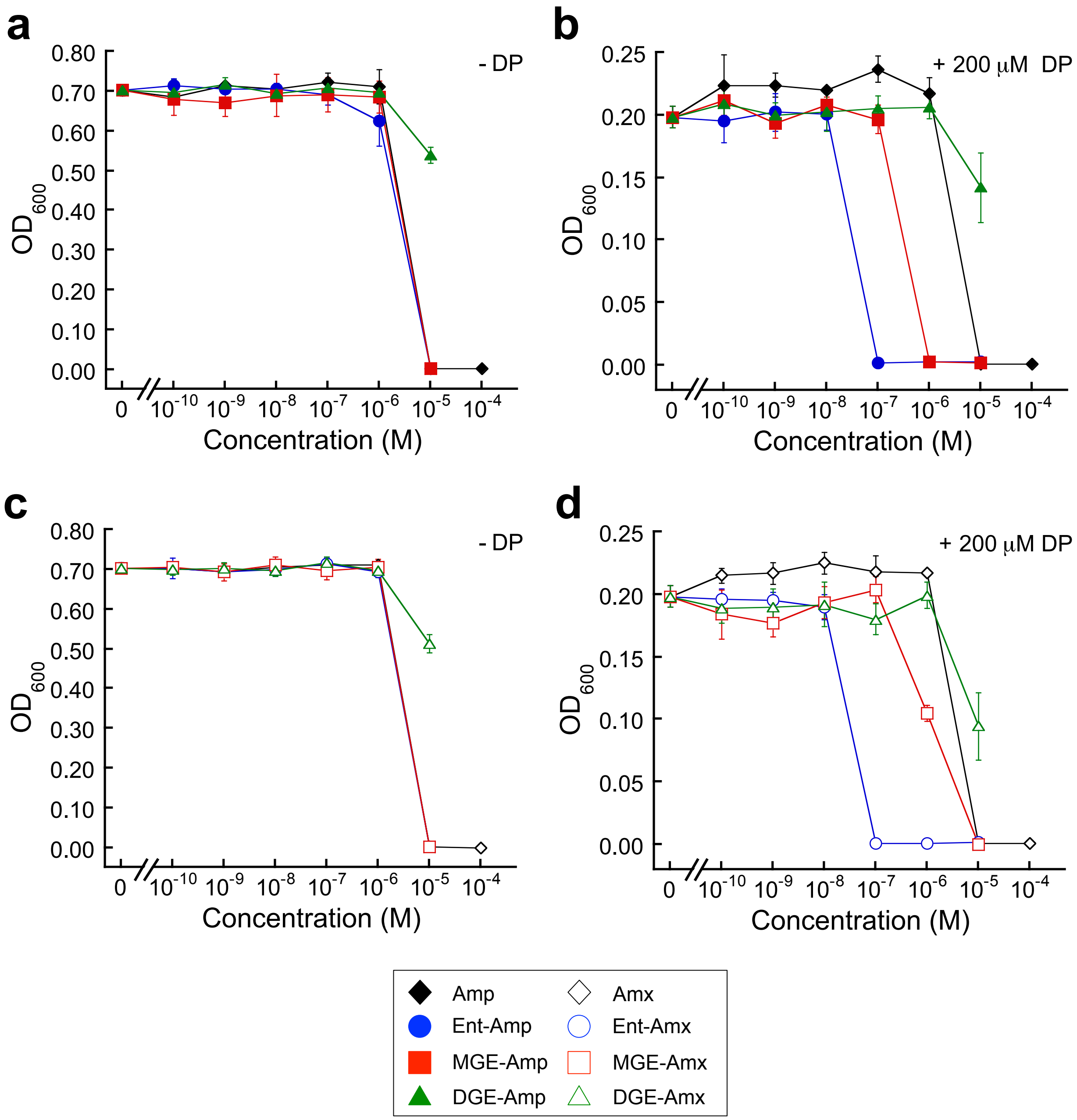

Fig. S7. Antibacterial activity of (Glc)Ent-Amp/Amx **5-10** against *E. coli* H9049 in 50% MHB medium in the absence and presence of 200 μM DP (t = 19 h, T = 30 °C) (mean ± standard deviation, n = 3). Panel b corresponds to Fig. 2c and is included for direct comparison.

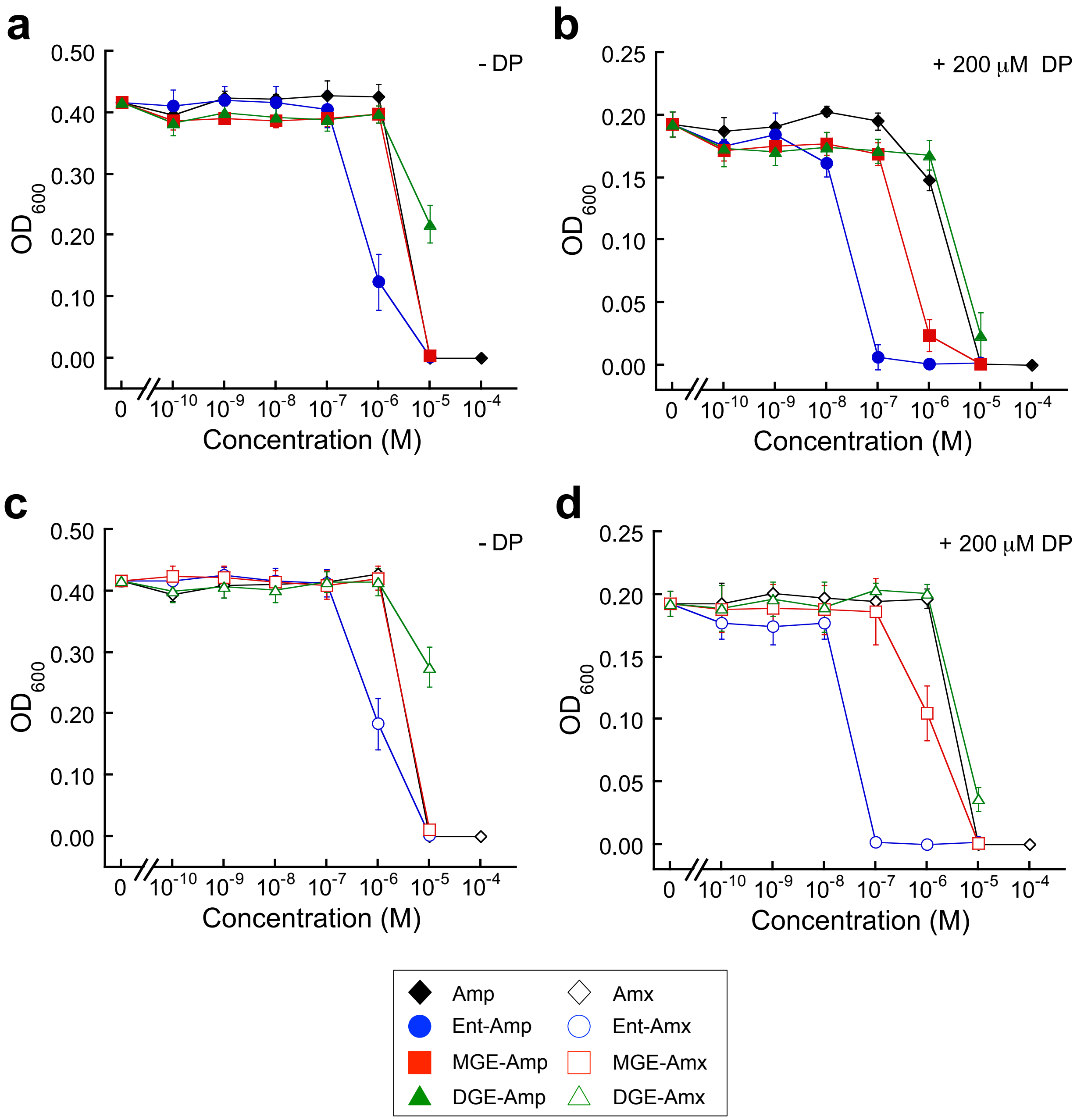

Fig. S8. Antibacterial activity of (Glc)Ent-Amp/Amx **5-10** against *E. coli* K-12 in 50% MHB medium in the absence and presence of 200 μM DP (t = 19 h, T = 30 °C) (mean ± standard deviation, n = 3). Panel b corresponds to Fig. 2d and is included for direct comparison.

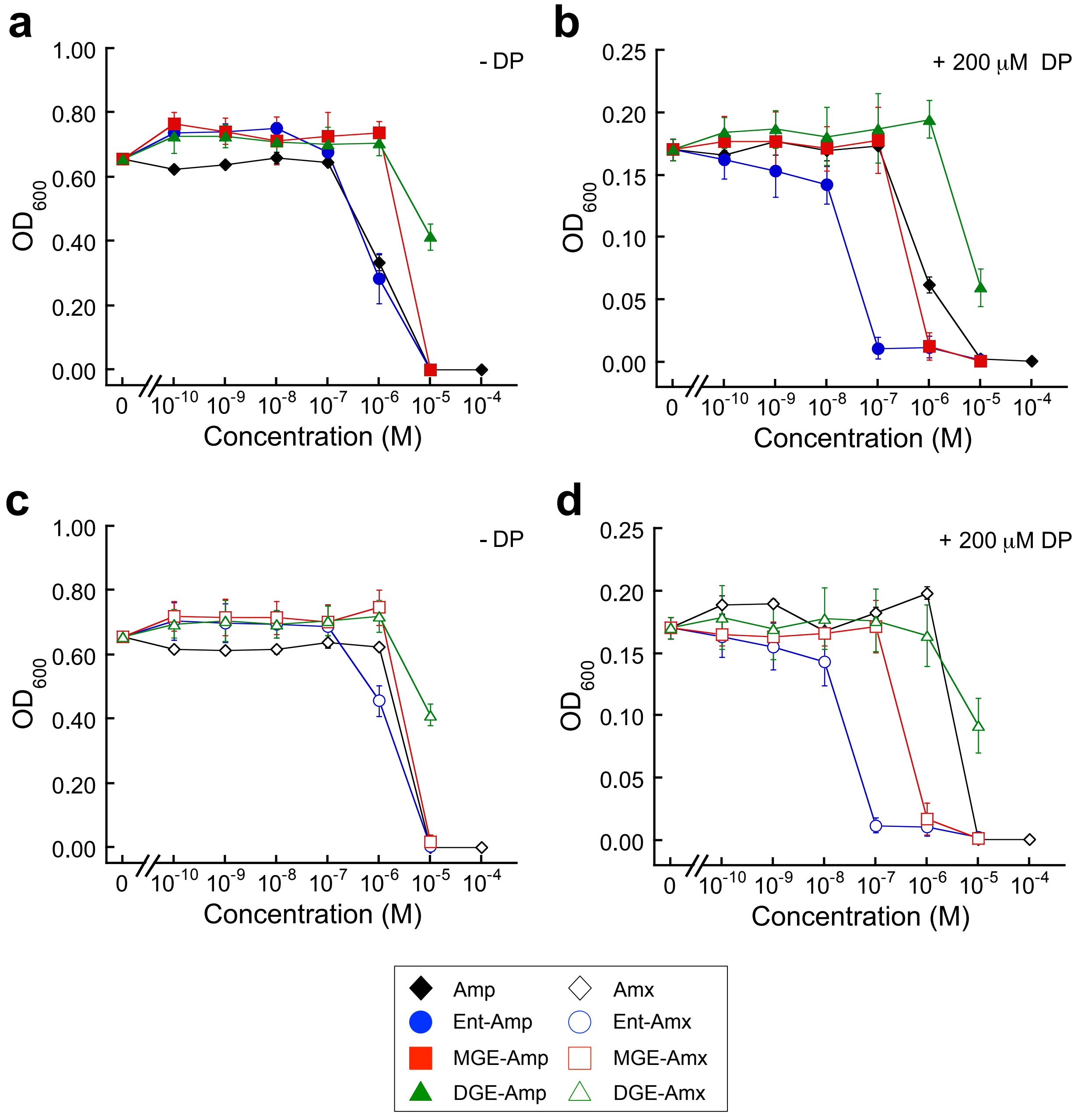

Fig. S9. Antibacterial activity of (Glc)Ent-Amp/Amx **5-10** against *E. coli* B in 50% MHB medium in the absence and presence of 200 μM DP (t = 19 h, T = 30 °C) (mean ± standard deviation, n = 3). Panel b corresponds to Fig. 2e and is included for direct comparison.

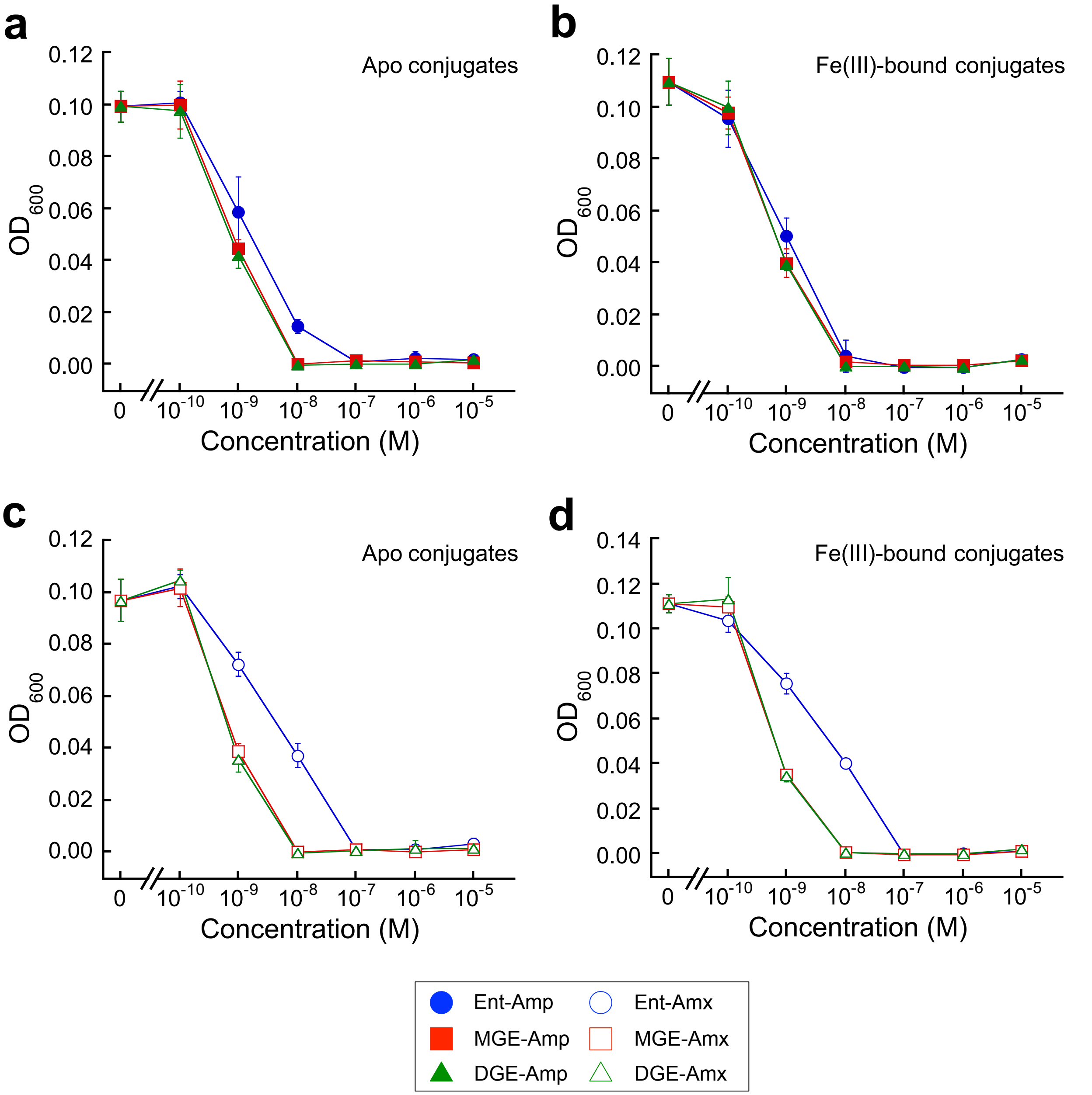

Fig. S10. Antibacterial activity of (a,c) apo or (b,d) Fe(III)-preloaded (0.95 equiv of iron) (Glc)Ent-Amp/Amx **5-10** against *E. coli* CFT073 in 50% MHB medium in the presence of 200 μM DP (t = 19 h, T = 30 °C) (mean ± standard deviation, n = 3).

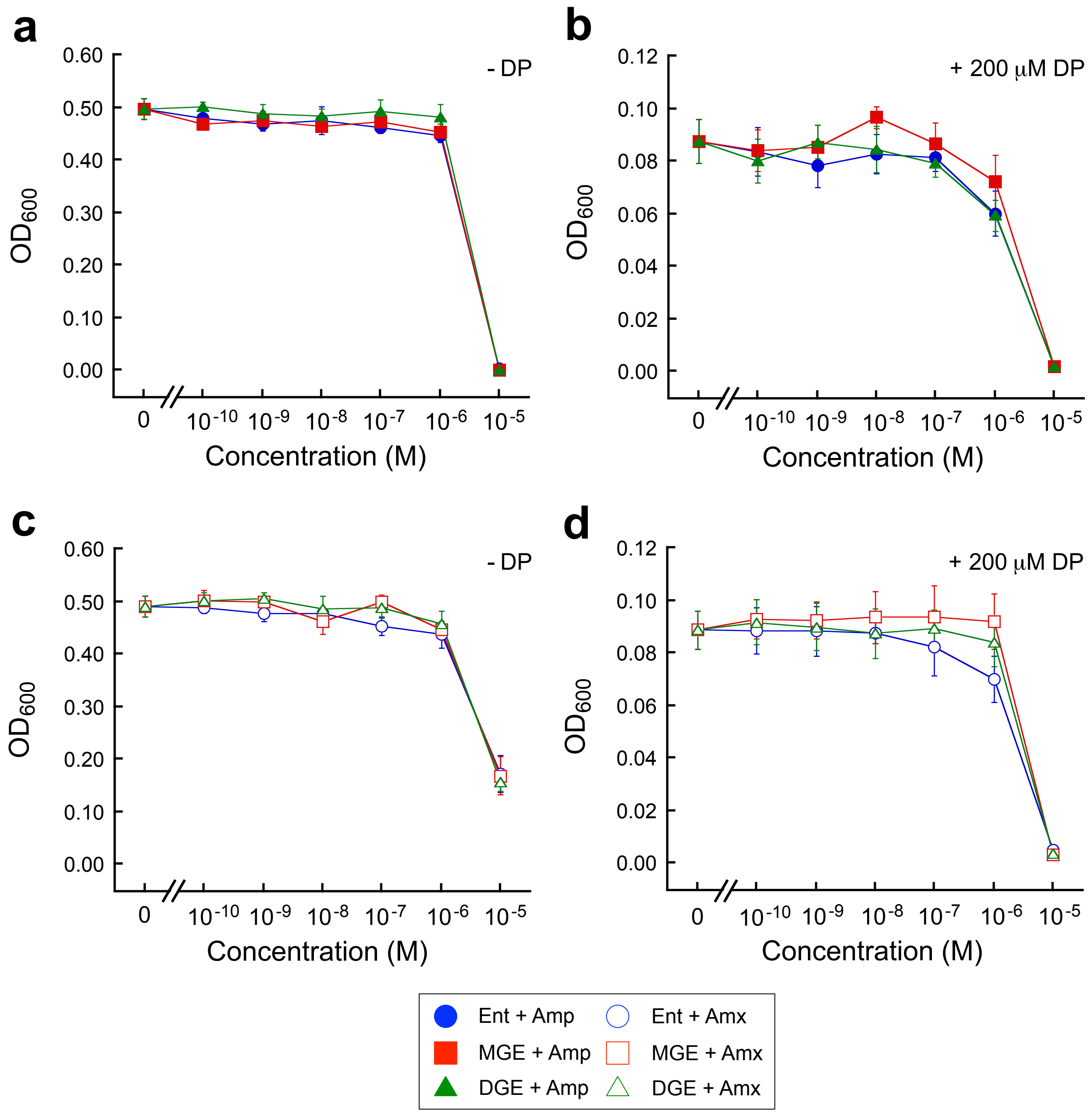

Fig. S11. Antibacterial activity of Amp, and Amx in the presence of exogenous Ent **1**, MGE **2**, or DGE **3** against *E. coli* CFT073 in 50% MHB medium in the absence and presence of 200 μM DP (t = 19 h, T = 30 °C) (mean ± standard deviation, n = 3).

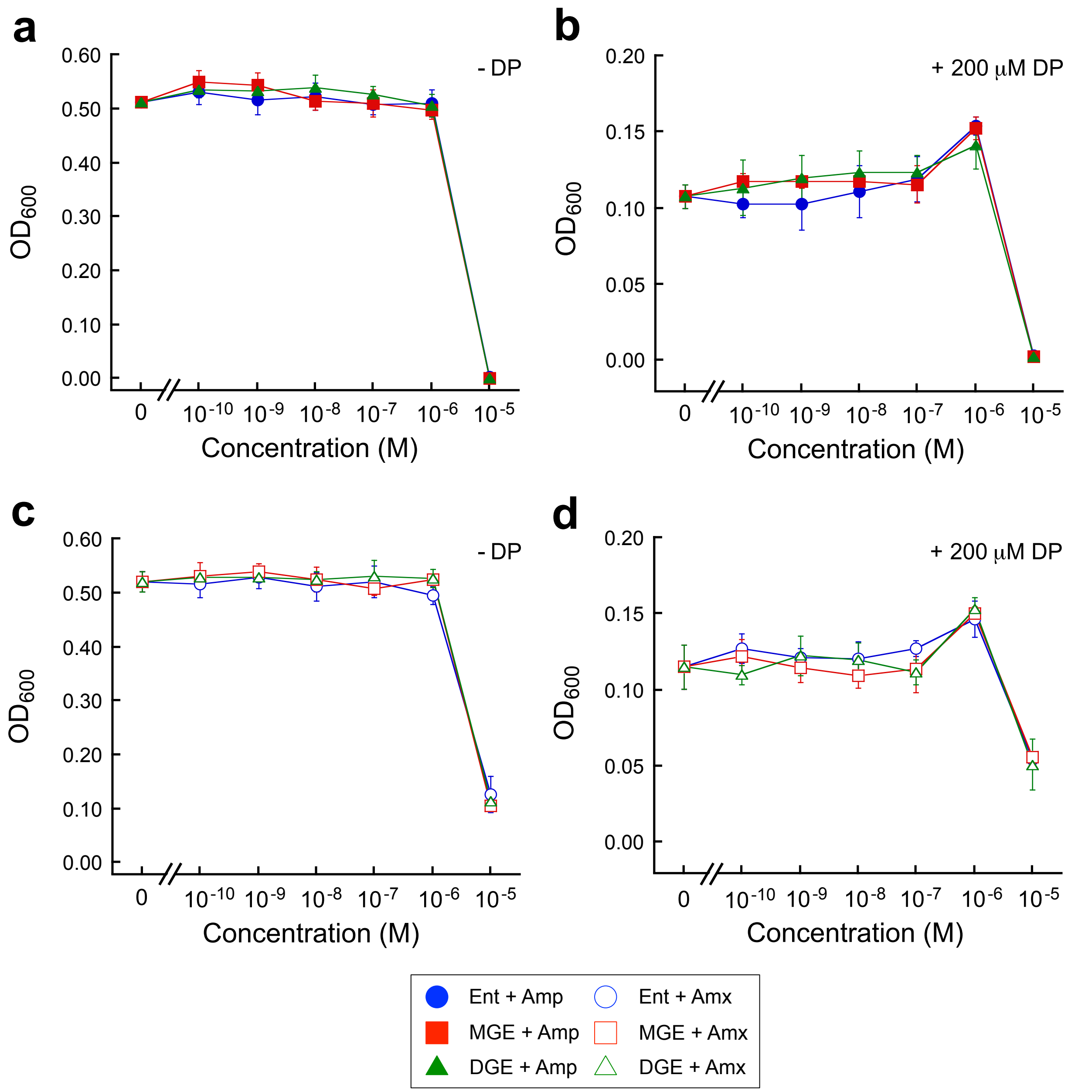

Fig. S12. Antibacterial activity of Amp, and Amx in the presence of exogenous Ent **1**, MGE **2**, or DGE **3** against *E. coli* UTI89 in 50% MHB medium in the absence and presence of 200 μM DP (t = 19 h, T = 30 °C) (mean ± standard deviation, n = 3).

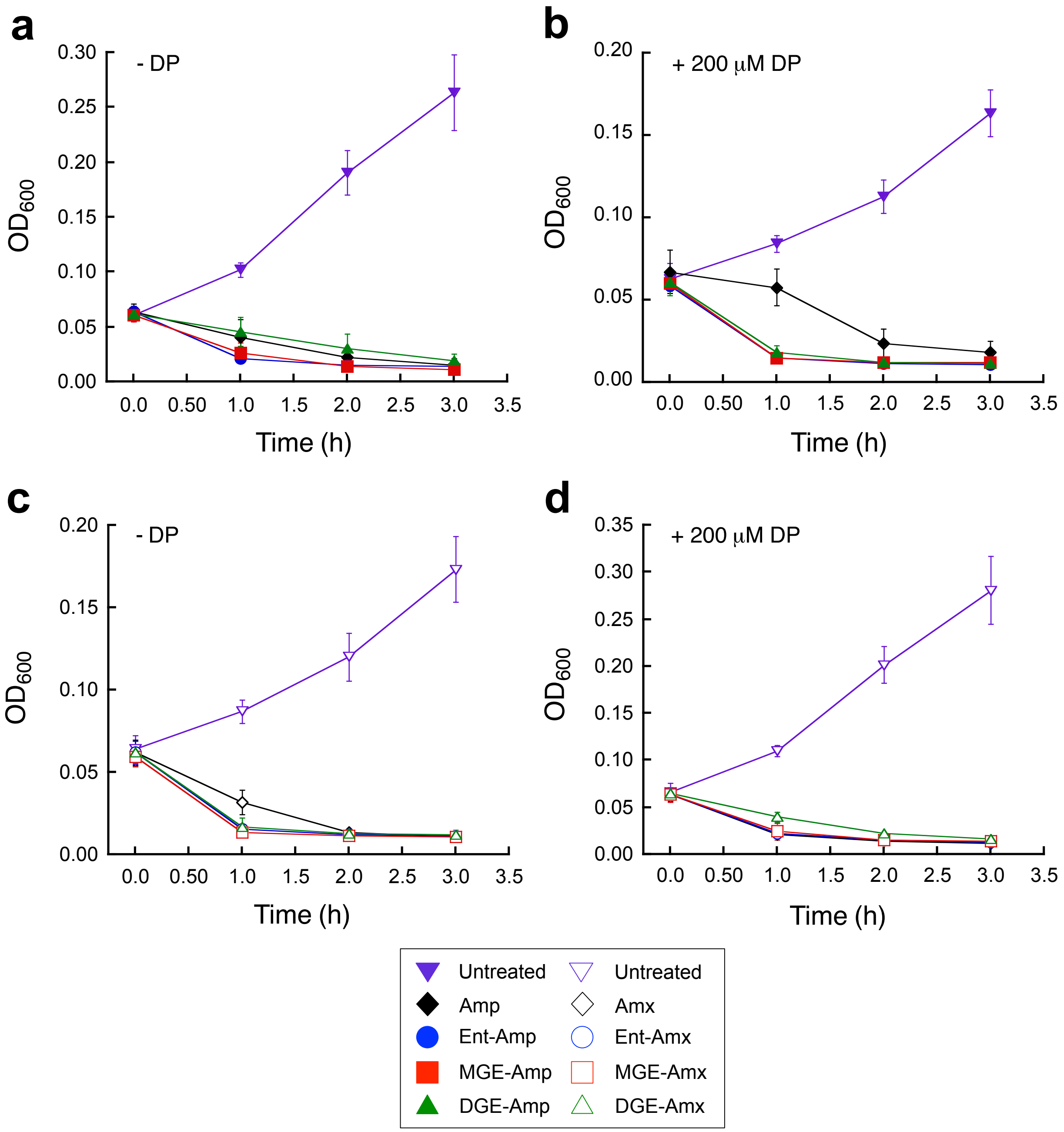

Fig. S13. Time-kill kinetics of (Glc)Ent-Amp/Amx **5-10** against *E. coli* CFT073 in 50% MHB medium in the absence and presence of 200 μM (T = 37 °C) (mean ± standard deviation, n = 3). (a,b) The bacteria (≈10^8 CFU/mL) are treated with 50 μM Amp or 5 μM (Glc)Ent-Amp. (c,d) The bacteria are treated with 50 μM Amx or 5 μM (Glc)Ent-Amx. Panel b corresponds to Fig. 3a and is included for direct comparison.

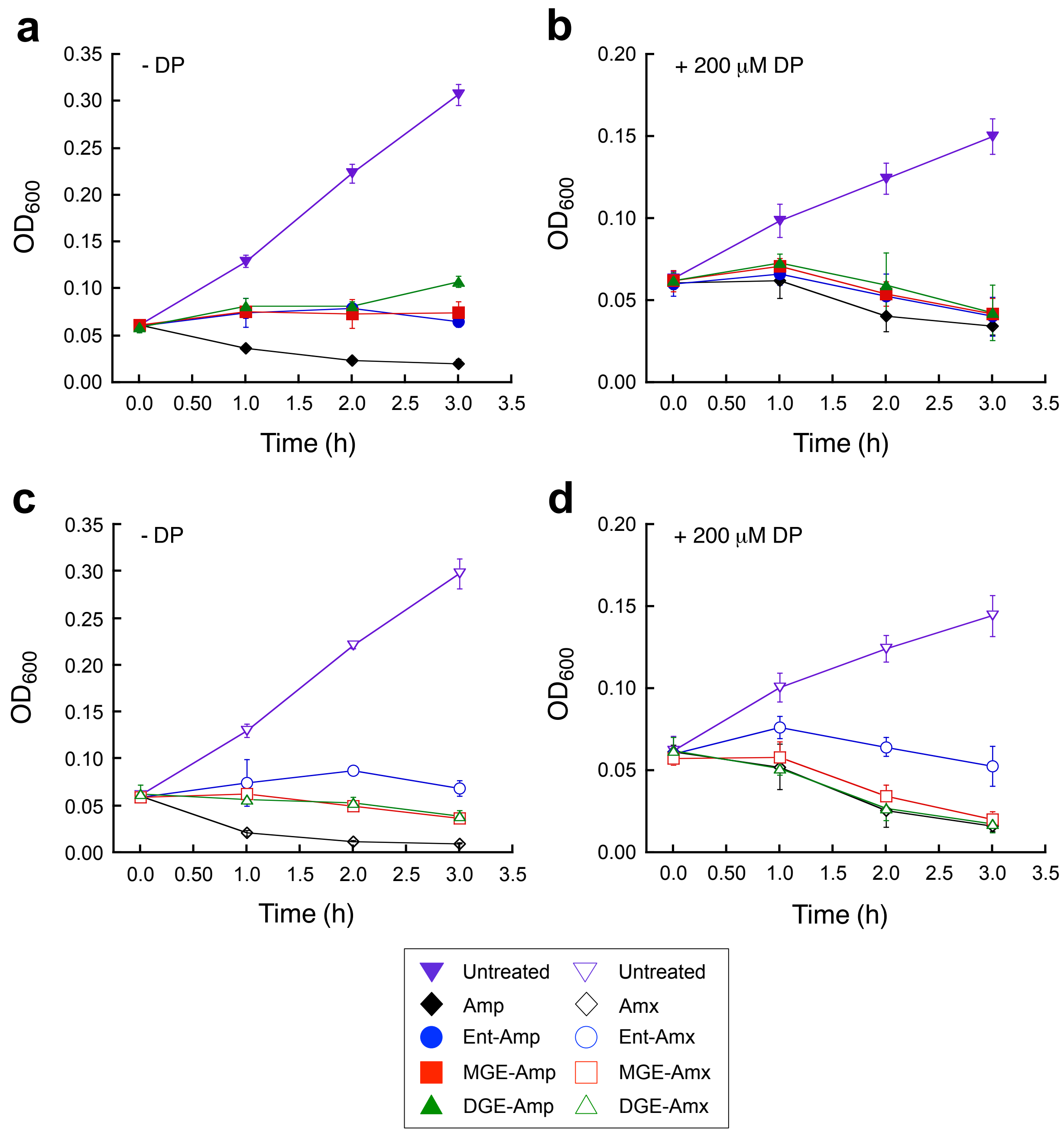

Fig. S14. Time-kill kinetics of (Glc)Ent-Amp/Amx **5-10** against *E. coli* UTI89 in 50% MHB medium in the absence and presence of 200 μM (T = 37 °C) (mean ± standard deviation, n = 3). (a,b) The bacteria (≈10^8 CFU/mL) are treated with 50 μM Amp or 50 μM (Glc)Ent-Amp. (c,d) The bacteria are treated with 50 μM Amx or 50 μM (Glc)Ent-Amx. Panel b corresponds to Fig. 3b and is included for direct comparison.

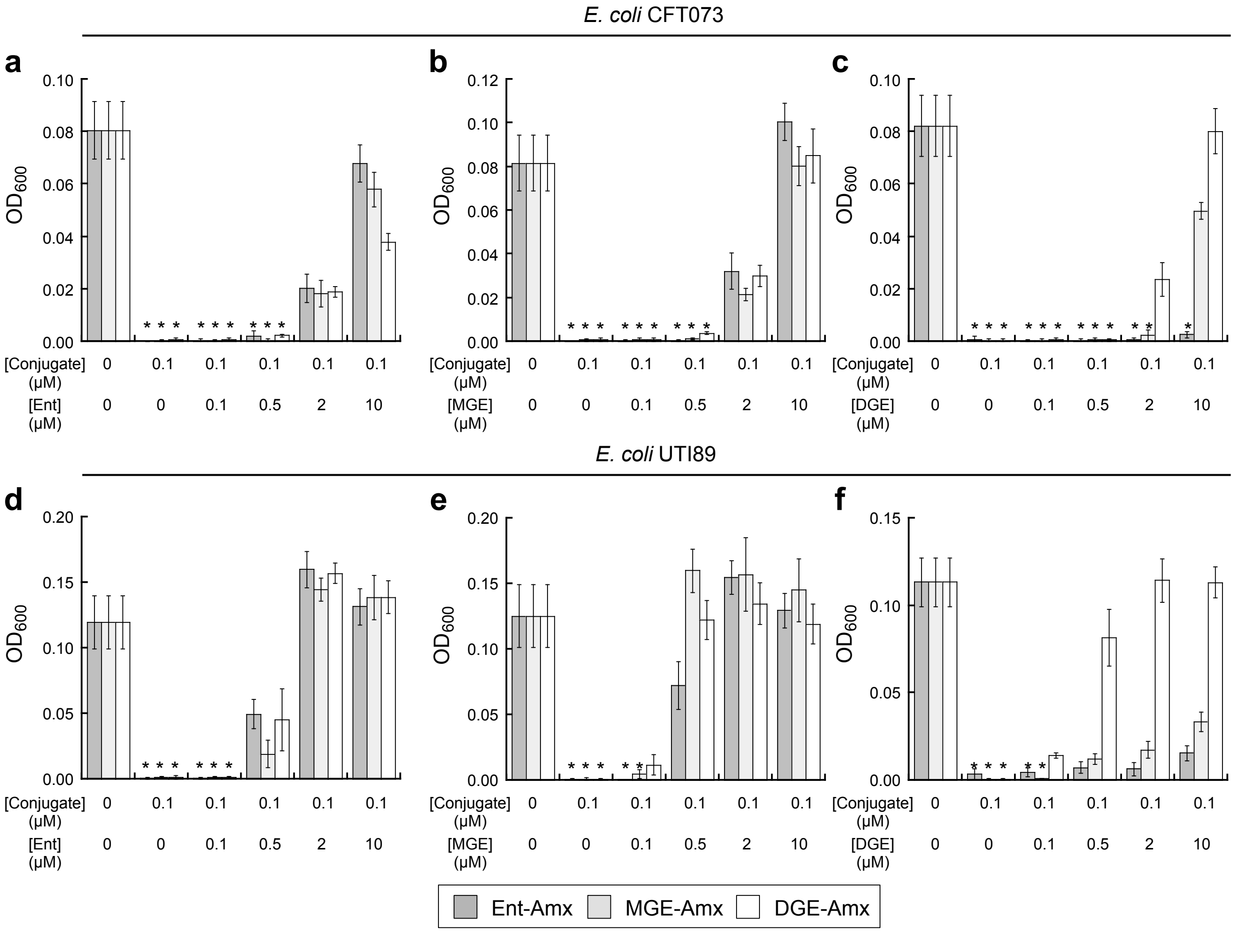

Fig. S15. (a)-(c) Growth of *E. coli* CFT073 in the presence of 100 nM (Glc)Ent-Amx **6/8/10** and mixtures of 100 nM (Glc)Ent-Amx **6/8/10** and 1, 5, 20, or 100 equiv of exogenous (a) Ent **1**, (b) MGE **2**, or (c) DGE **3** in the presence of 200 μM DP. (d)-(f) Growth of *E. coli* UTI89 in the presence of 100 nM (Glc)Ent-Amx **6/8/10** and mixtures of 100 nM (Glc)Ent-Amx **6/8/10** and 1, 5, 20, or 100 equiv of exogenous (d) Ent **1**, (e) MGE **2**, or (f) DGE **3** in the presence of 200 μM DP. All assays were performed in 50% MHB medium (t = 19 h, T = 30 °C) (mean ± standard deviation, n = 3). An asterisk indicates OD_{600} < 0.01.

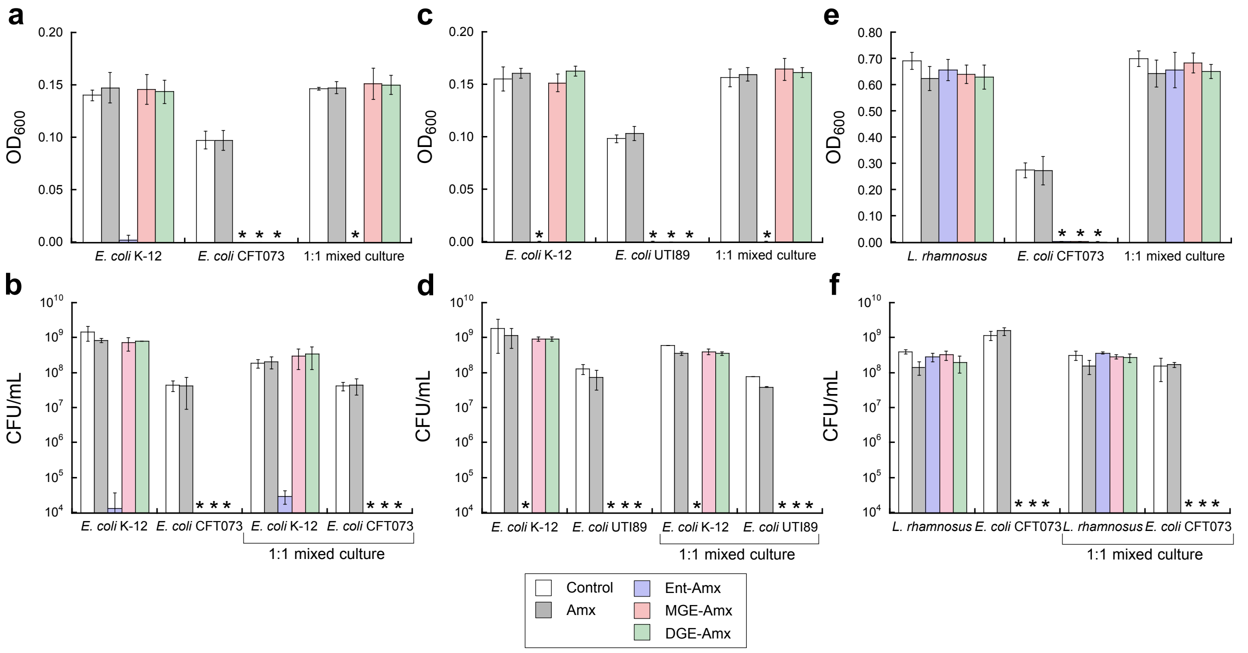

Fig. S16. (a,b) Bacterial growth monitored by (a) OD_{600} and (b) CFU/mL for cultures of *E. coli* K-12 only, CFT073 only, and 1:1 K-12/CFT073 mixtures treated with 100 nM Amx or 100 nM (Glc)Ent-Amx **6/8/10** in the presence of 200 μM DP. (c,d) Bacterial growth monitored by (c) OD_{600} and (d) CFU/mL for cultures of *E. coli* K-12 only, UTI89 only, and 1:1 K-12/UTI89 mixtures treated with 100 nM Amx or 100 nM (Glc)Ent-Amx **6/8/10** in the presence of 200 μM DP. (e,f) Bacterial growth monitored by (e) OD_{600} and (f) CFU/mL for cultures of *L. rhamnosus* GG ATCC 53103 only, *E. coli* CFT073 only, and 1:1 *L. rhamnosus* GG/*E. coli* CFT073 mixtures treated with 1 μM Amx or 1 μM (Glc)Ent-Amx **6/8/10** in the presence of 200 μM DP. All mixed-*E. coli* antimicrobial assays were performed in 50% MHB medium and all mixed-species antimicrobial assays were conducted in 1:1 MRS/MHB medium (t = 19 h, T = 30 °C) (mean ± standard deviation, n = 3). An asterisk indicates $OD_{600} < 0.01$ or no colony formation.

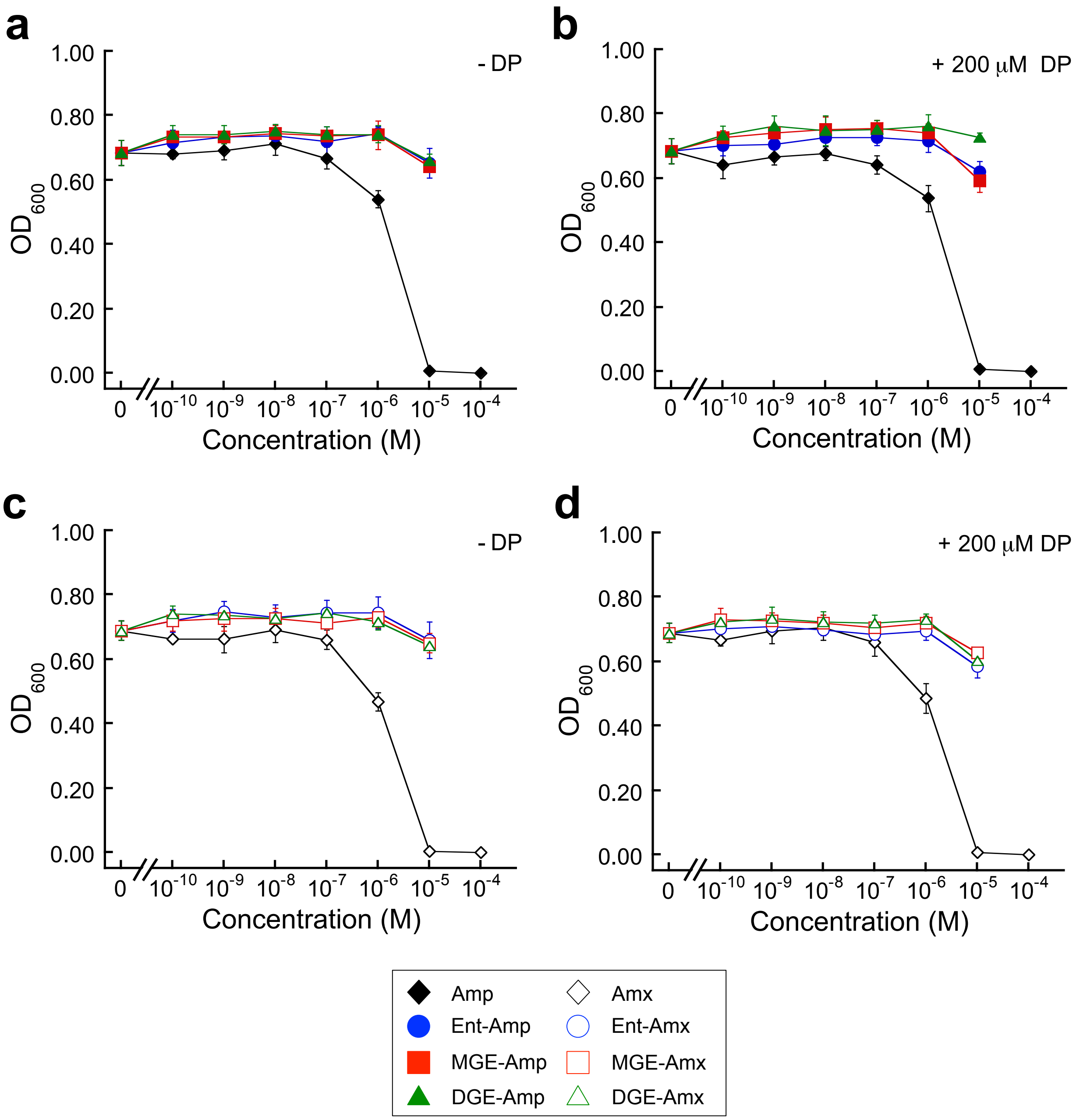

Fig. S17. Antibacterial activity of (Glc)Ent-Amp/Amx **5-10** against *L. rhamnosus* GG (ATCC 53103) in 1:1 MRS/MHB medium in the absence and presence of 200 μM DP (t = 19 h, T = 30 °C) (mean ± standard deviation, n = 3).

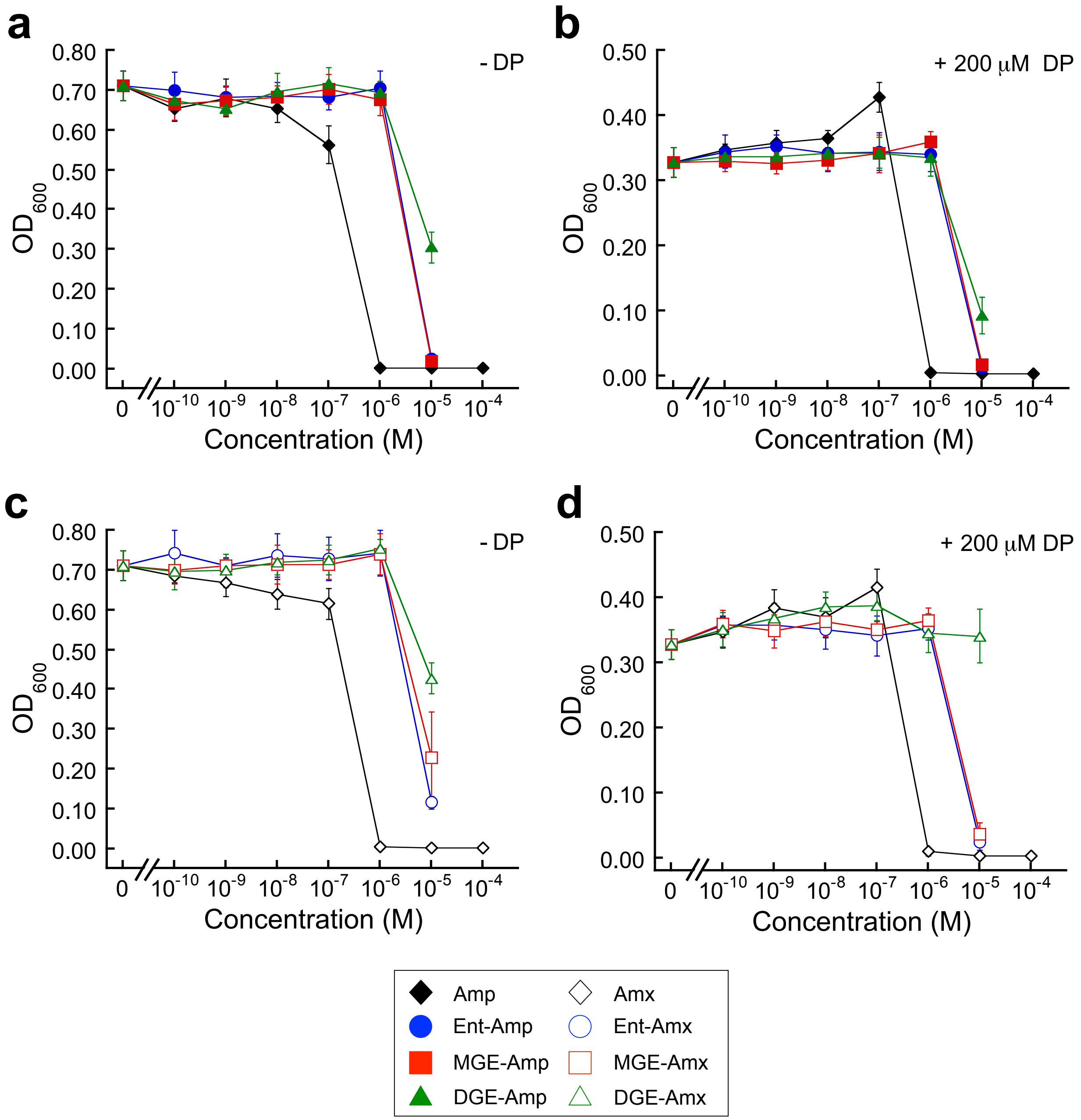

Fig. S18. Antibacterial activity of (Glc)Ent-Amp/Amx **5-10** against *S. aureus* ATCC 25923 in 50% MHB medium in the absence and presence of 200 μM DP (t = 19 h, T = 30 °C) (mean ± standard deviation, n = 3).

Note: These data are consistent with our prior report of Ent-Amp/Amx antibacterial activity against *S. aureus* ATCC 25923 (ref. 1 of Supplementary Information).

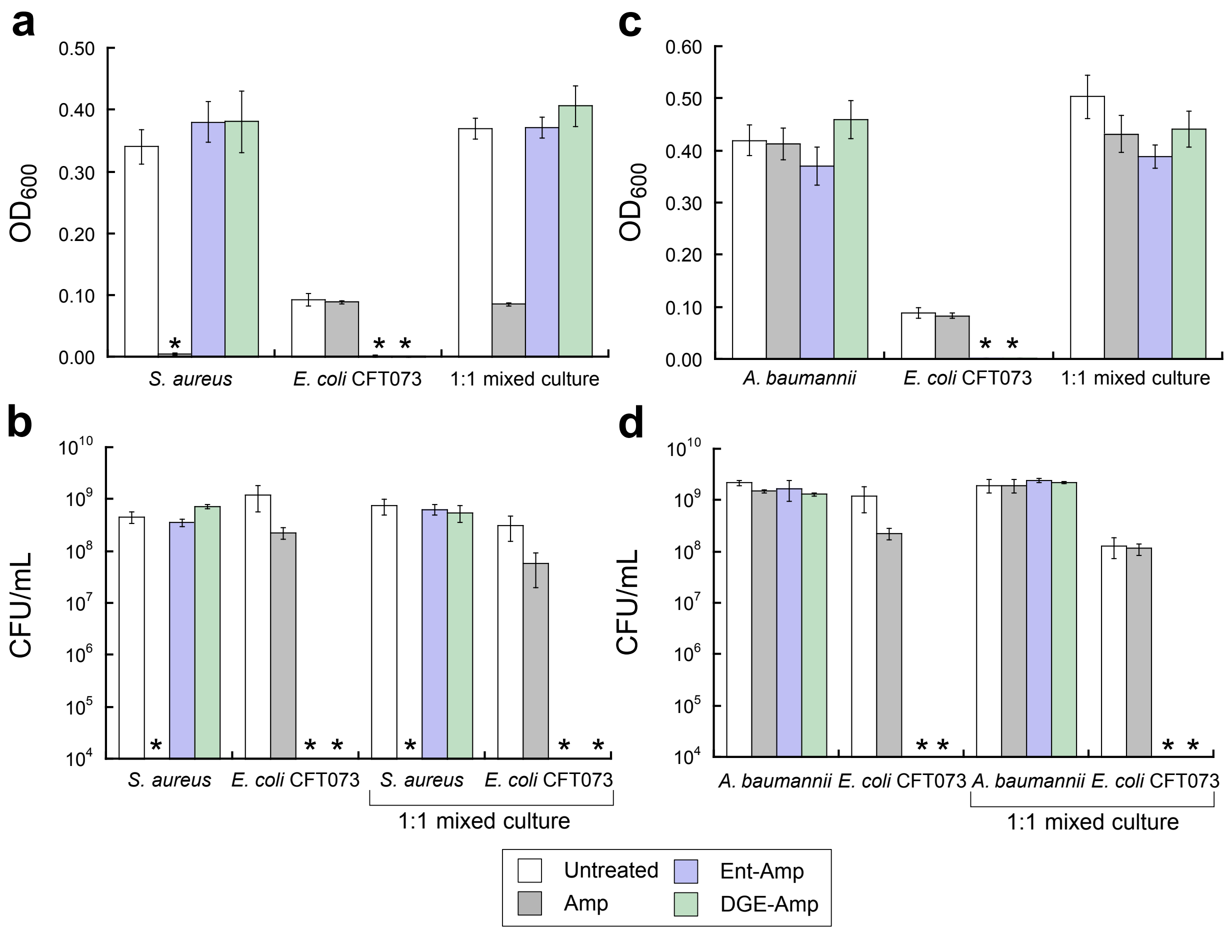

Fig. S19. (a,b) Bacterial growth monitored by (a) OD_{600} and (b) CFU/mL for cultures of *S. aureus* ATCC 25923 only, *E. coli* CFT073 only, and 1:1 *S. aureus* / *E. coli* CFT073 mixtures treated with 1 μM Amp or 1 μM (Glc)Ent-Amp **5/7/9** in the presence of 200 μM DP. (c,d) Bacterial growth monitored by (c) OD_{600} and (d) CFU/mL for cultures of *A. baumannii* ATCC 17961 only, *E. coli* CFT073 only, and 1:1 *A. baumannii* / *E. coli* CFT073 mixtures treated with 1 μM Amp or 1 μM (Glc)Ent-Amp **5/7/9** in the presence of 200 μM DP. These assays were performed in 50% MHB medium (t = 19 h, T = 30 °C) (mean ± standard deviation, n = 3). An asterisk indicates OD_{600} < 0.01 or no colony formation.

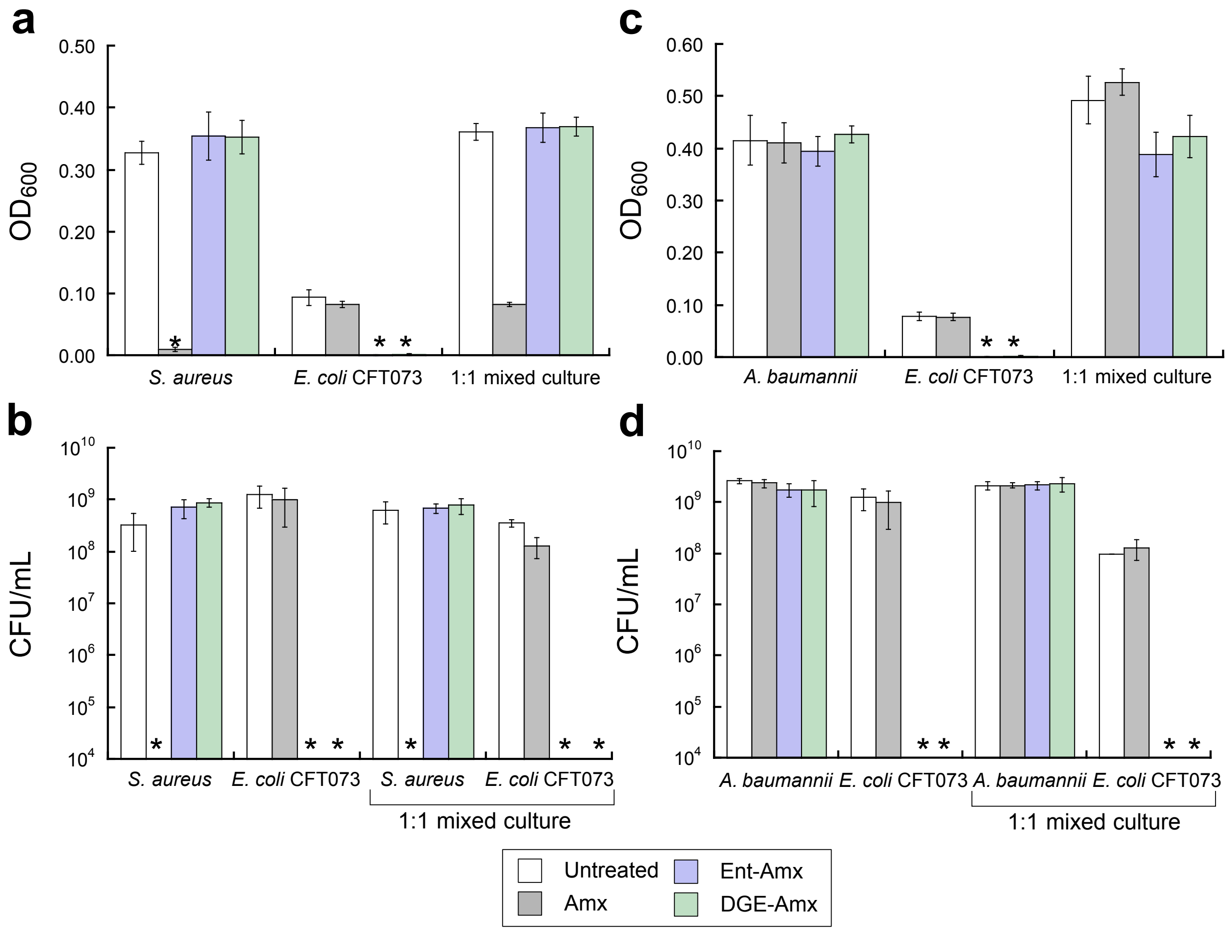

Fig. S20. (a,b) Bacterial growth monitored by (a) OD_{600} and (b) CFU/mL for cultures of *S. aureus* ATCC 25923 only, *E. coli* CFT073 only, and 1:1 *S. aureus* / *E. coli* CFT073 mixtures treated with 1 μM Amx or 1 μM (Glc)Ent-Amx **6/8/10** in the presence of 200 μM DP. (c,d) Bacterial growth monitored by (c) OD_{600} and (d) CFU/mL for cultures of *A. baumannii* ATCC 17961 only, *E. coli* CFT073 only, and 1:1 *A. baumannii* / *E. coli* CFT073 mixtures treated with 1 μM Amx or 1 μM (Glc)Ent-Amx **6/8/10** in the presence of 200 μM DP. These assays were performed in 50% MHB medium (t = 19 h, T = 30 °C) (mean ± standard deviation, n = 3). An asterisk indicates OD_{600} < 0.01 or no colony formation.

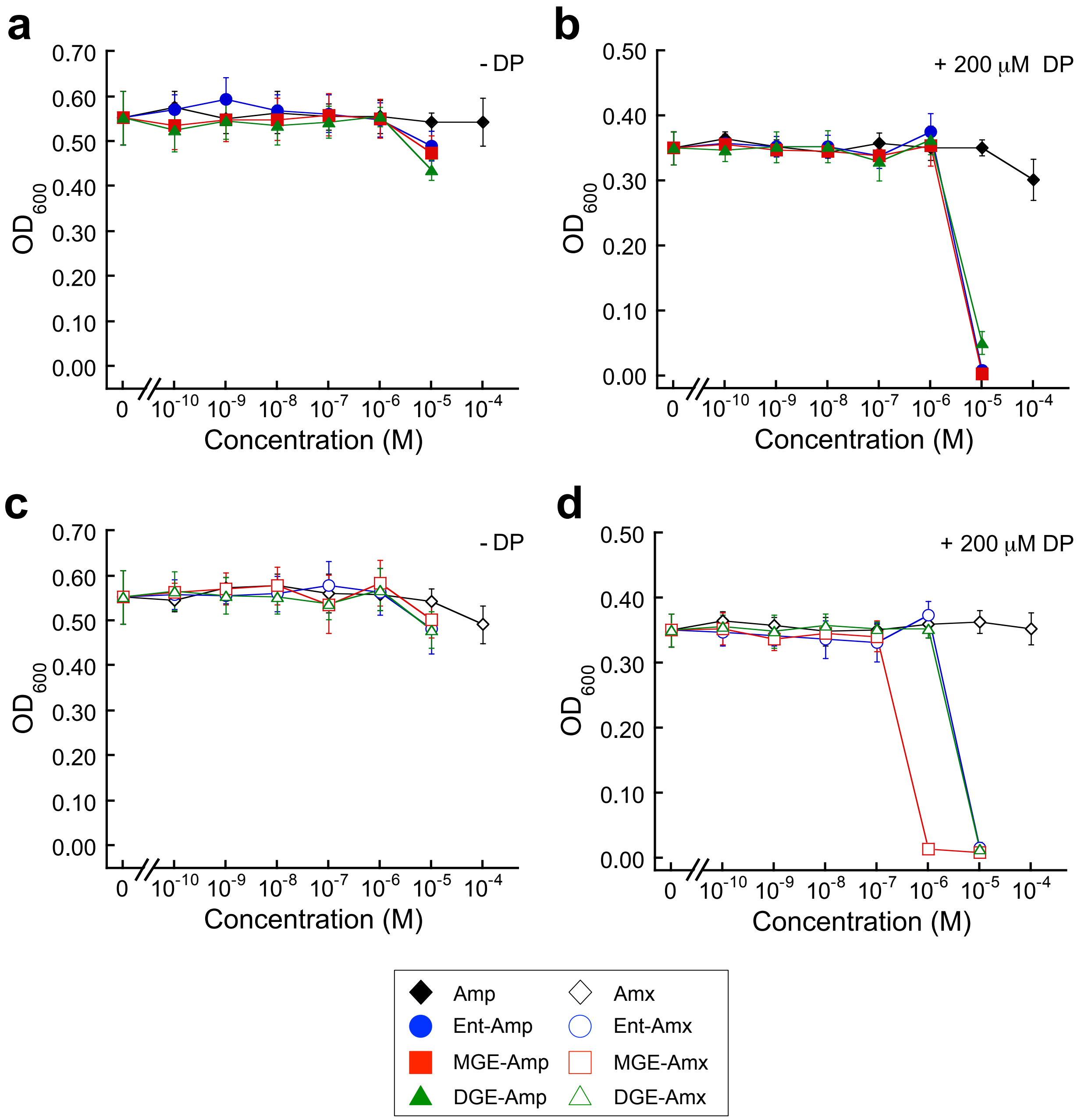

Fig. S21. Antibacterial activity of (Glc)Ent-Amp/Amx **5-10** against *A. baumannii* ATCC 17961 in 50% MHB medium in the absence and presence of 200 μM DP (t = 19 h, T = 30 °C) (mean ± standard deviation, n = 3).

Note: This *A. baumannii* strain is insensitive to Amp/Amx over the concentration range tested. The activity observed for the conjugates in the presence of DP likely arises from iron deprivation caused by the conjugates sequestering Fe(III) in the growth medium. The origin of the enhanced activity of MGE-Amx against *A. baumannii* (+DP) is as-yet undetermined.

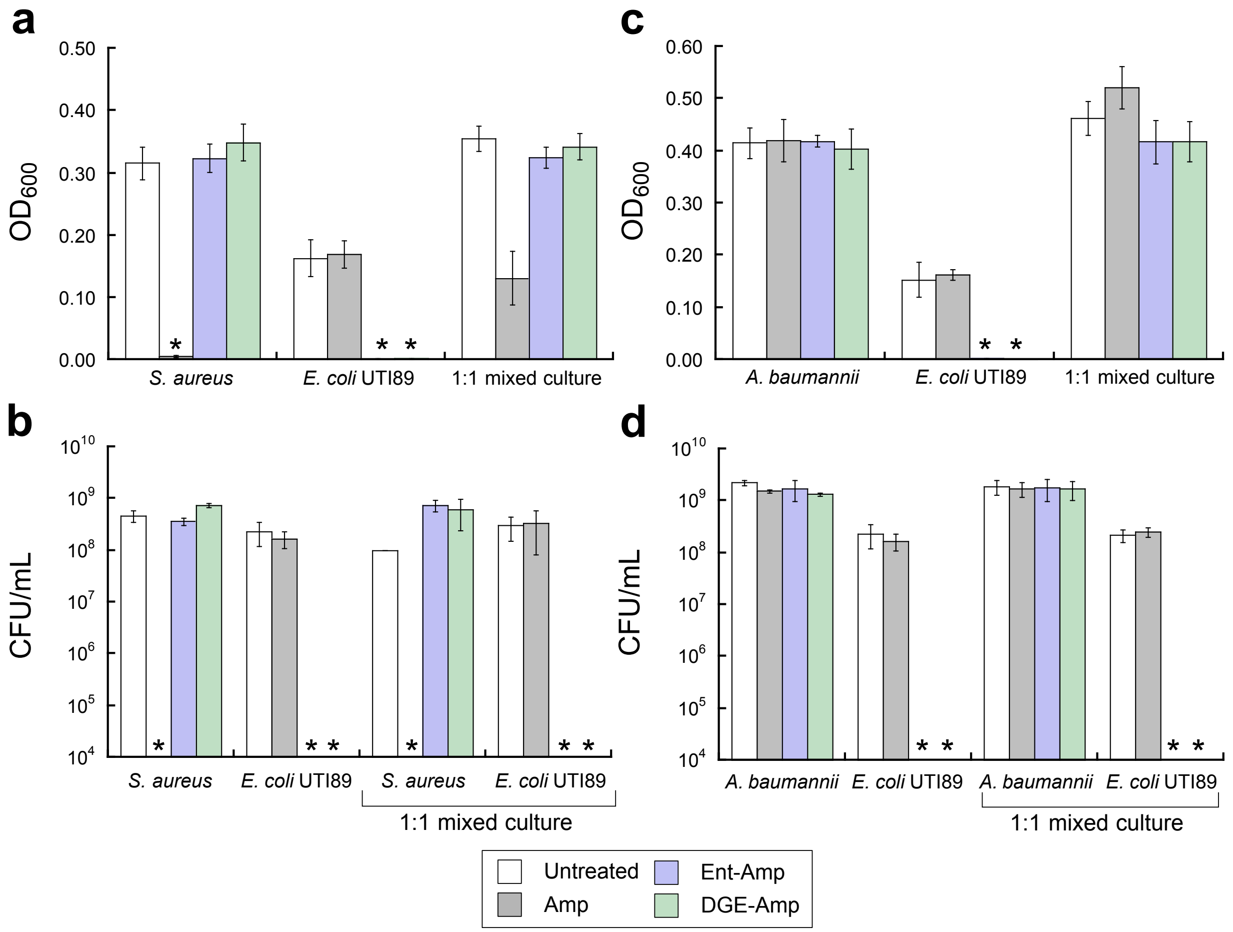

Fig. S22. (a,b) Bacterial growth monitored by (a) OD_{600} and (b) CFU/mL for cultures of *S. aureus* ATCC 25923 only, *E. coli* UTI89 only, and 1:1 *S. aureus* / *E. coli* UTI89 mixtures treated with 1 μM Amp or 1 μM (Glc)Ent-Amp **5/7/9** in the presence of 200 μM DP. (c,d) Bacterial growth monitored by (c) OD_{600} and (d) CFU/mL for cultures of *A. baumannii* ATCC 17961 only, *E. coli* UTI89 only, and 1:1 *A. baumannii* / *E. coli* UTI89 mixtures treated with 1 μM Amp or 1 μM (Glc)Ent-Amp **5/7/9** in the presence of 200 μM DP. These assays were performed in 50% MHB medium (t = 19 h, T = 30 °C) (mean ± standard deviation, n = 3). An asterisk indicates OD_{600} < 0.01 or no colony formation.

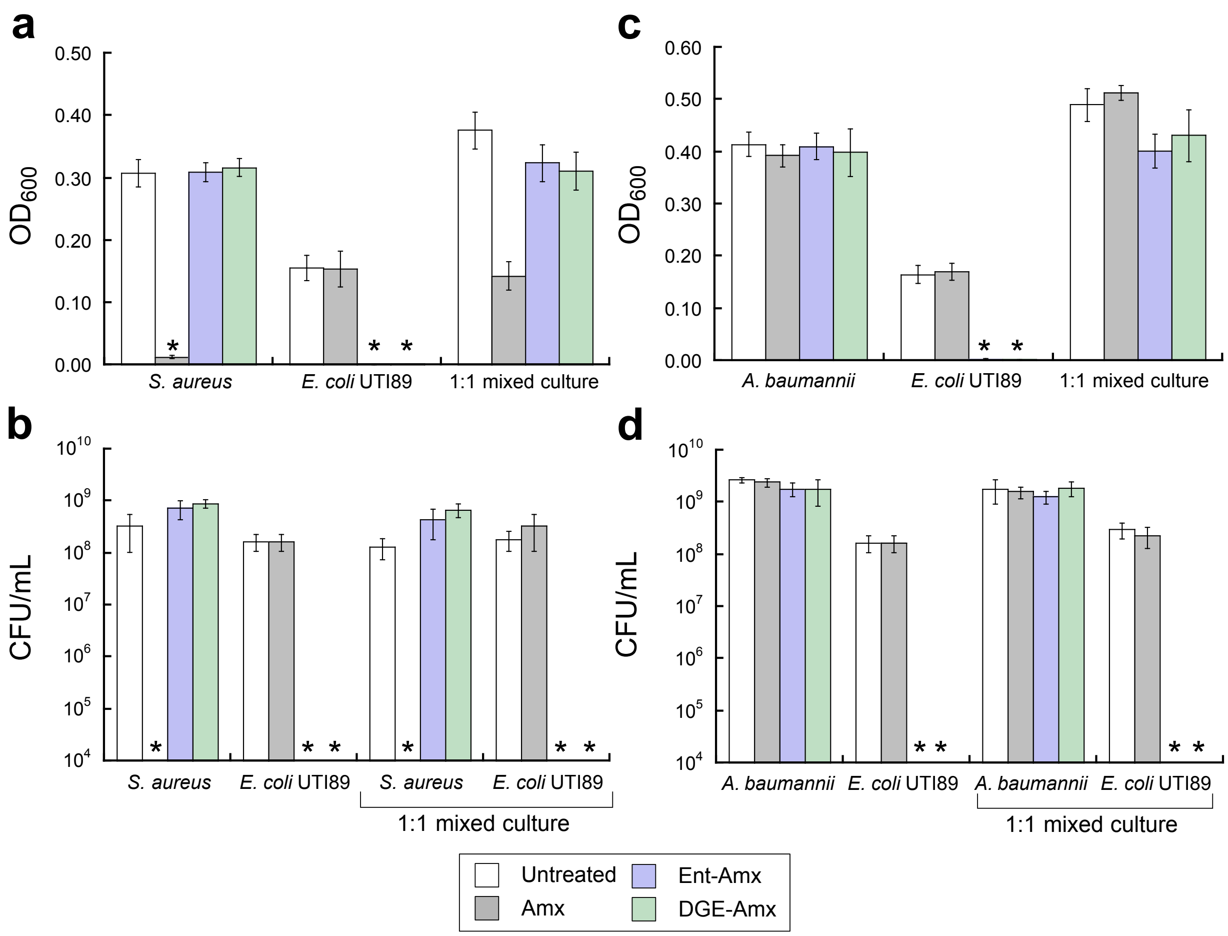

Fig. S23. (a,b) Bacterial growth monitored by (a) OD_{600} and (b) CFU/mL for cultures of *S. aureus* ATCC 25923 only, *E. coli* UTI89 only, and 1:1 *S. aureus* / *E. coli* UTI89 mixtures treated with 1 μM Amx or 1 μM (Glc)Ent-Amx **6/8/10** in the presence of 200 μM DP. (c,d) Bacterial growth monitored by (c) OD_{600} and (d) CFU/mL for cultures of *A. baumannii* ATCC 17961 only, *E. coli* UTI89 only, and 1:1 *A. baumannii* / *E. coli* UTI89 mixtures treated with 1 μM Amx or 1 μM (Glc)Ent-Amx **6/8/10** in the presence of 200 μM DP. These assays were performed in 50% MHB medium (t = 19 h, T = 30 °C) (mean ± standard deviation, n = 3). An asterisk indicates OD_{600} < 0.01 or no colony formation.

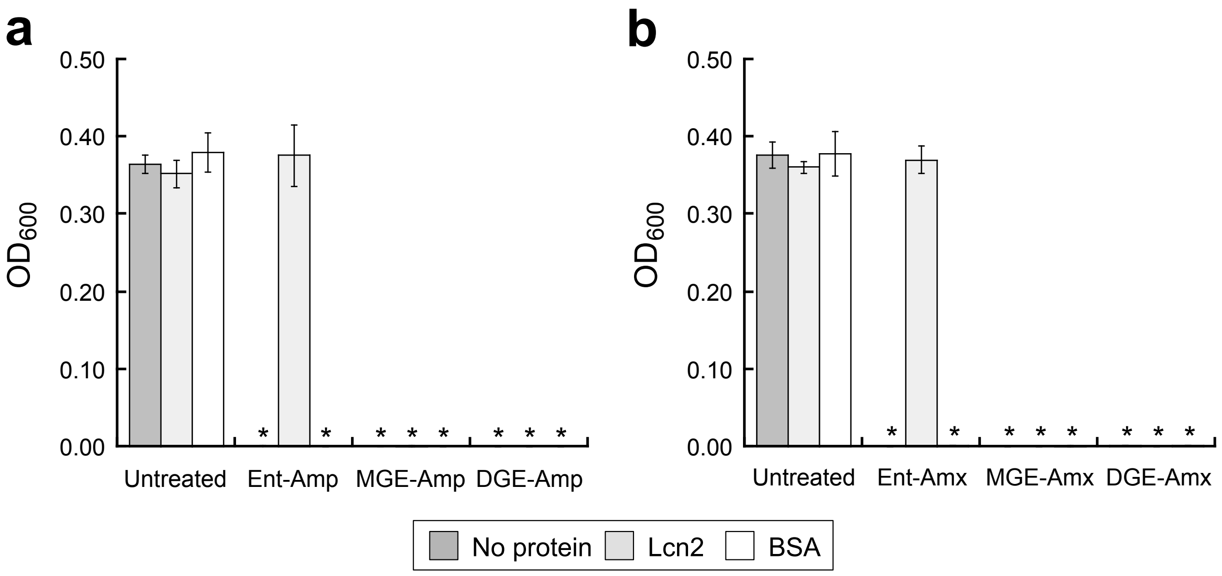

Fig. S24. Antibacterial activity of (Glc)Ent-Amp/Amx **5-10** against *E. coli* CFT073 in the presence of Lcn2 or BSA. *E. coli* CFT073 was treated with (a) 100 nM ferric-preloaded (Glc)Ent-Amp **5/7/9** or (b) 100 nM ferric-preloaded (Glc)Ent-Amx **6/8/10** in the absence and presence of 1 μM Lcn2 or 1 μM BSA. For Fe(III) preloading, 0.95 equiv of Fe(III) was added to each conjugate. The assays were conducted in modified M9 medium (t = 24 h, T = 37 °C) (mean ± standard deviation, n = 3). An asterisk indicates OD_{600} < 0.01.

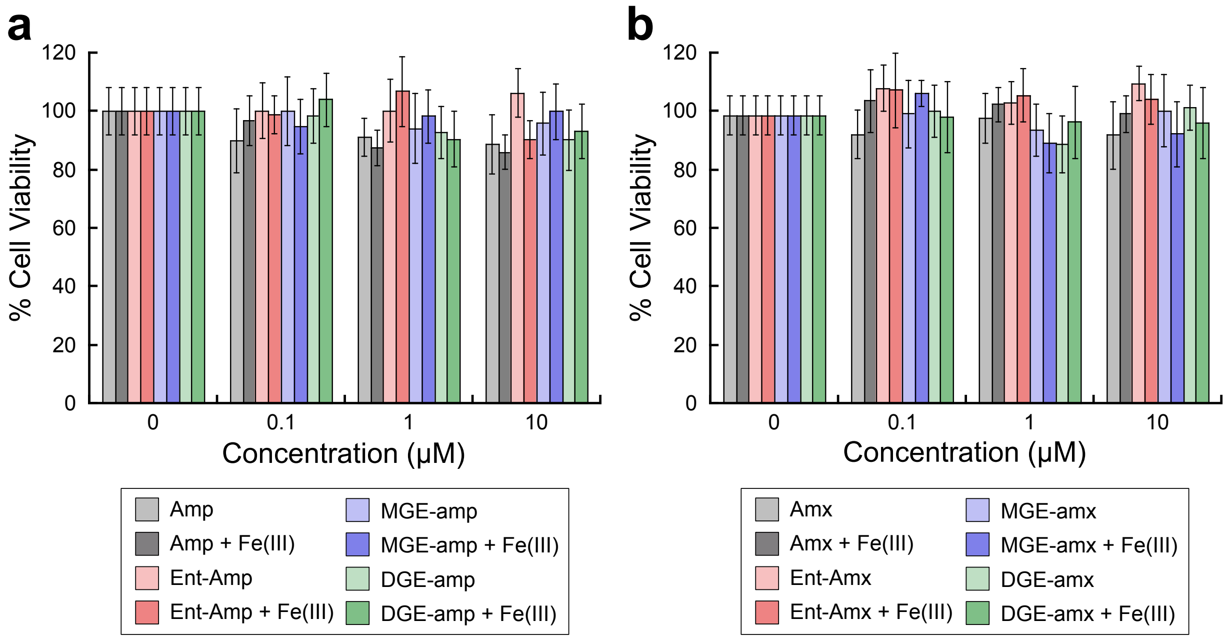

Fig. S25. Cytotoxicity studies of apo and Fe(III)-preloaded (Glc)Ent-Amp/Amx **5-10** against human colonic epithelial cells (T84 cells) in 1:1 DMEM/F-12 with 10% FBS, and 1% penicillin and streptomycin (t = 24 h, T = 37 °C, 5% CO_2). Percentage of cell viability is quantified by MTT assay after a 24-h treatment of the conjugates (mean ± standard deviation, n = 3).

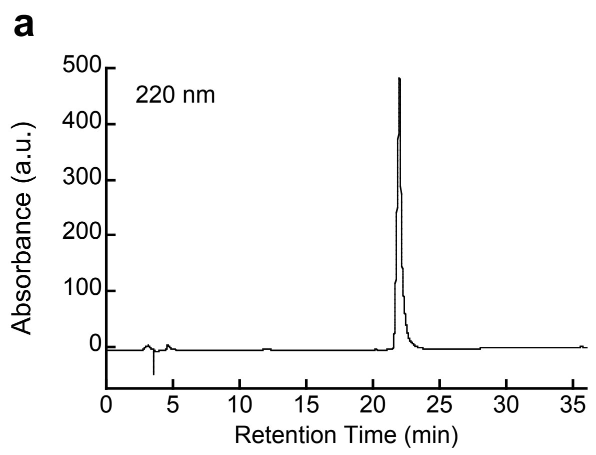

Fig. S26. Analytical HPLC traces of purified MGE-Amp **7** (0% B for 5 min followed by 0-100% B over 30 min, 1 mL/min). (a) Absorbance monitored at 220 nm. (b) Absorbance monitored at 316 nm. The sample was dissolved in 1:1 MeCN/H_2O.

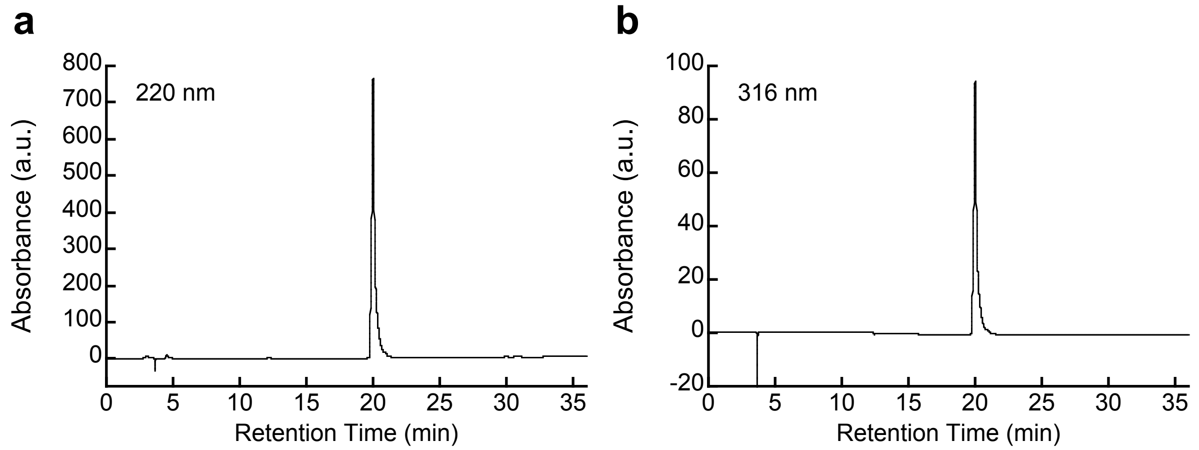

Fig. S27. Analytical HPLC traces of purified MGE-Amx **8** (0% B for 5 min followed by 0-100% B over 30 min, 1 mL/min). (a) Absorbance monitored at 220 nm. (b) Absorbance monitored at 316 nm. The sample was dissolved in 1:1 MeCN/H_2O.

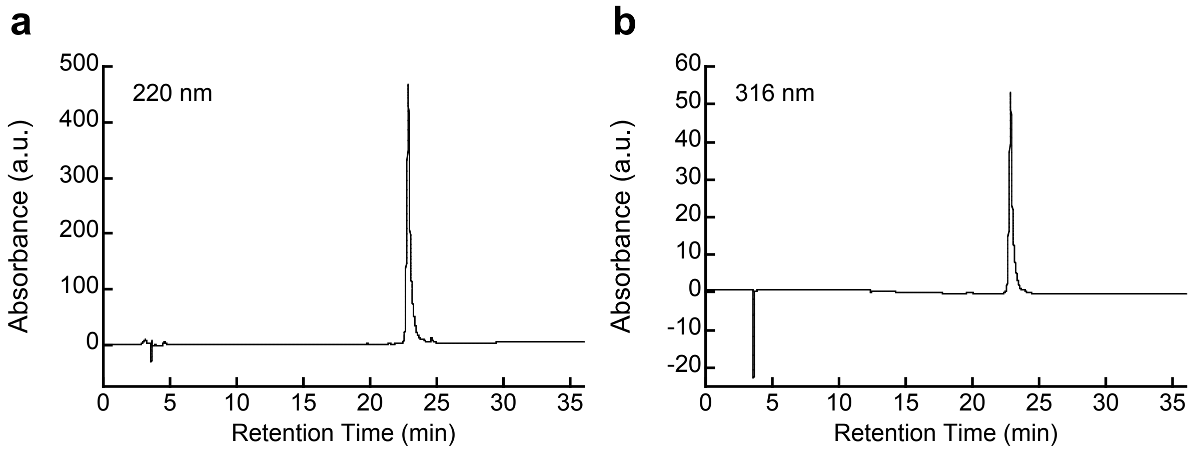

Fig. S28. Analytical HPLC traces of purified DGE-Amp **9** (0% B for 5 min followed by 0-100% B over 30 min, 1 mL/min). (a) Absorbance monitored at 220 nm. (b) Absorbance monitored at 316 nm. The sample was dissolved in 1:1 MeCN/H_2O.

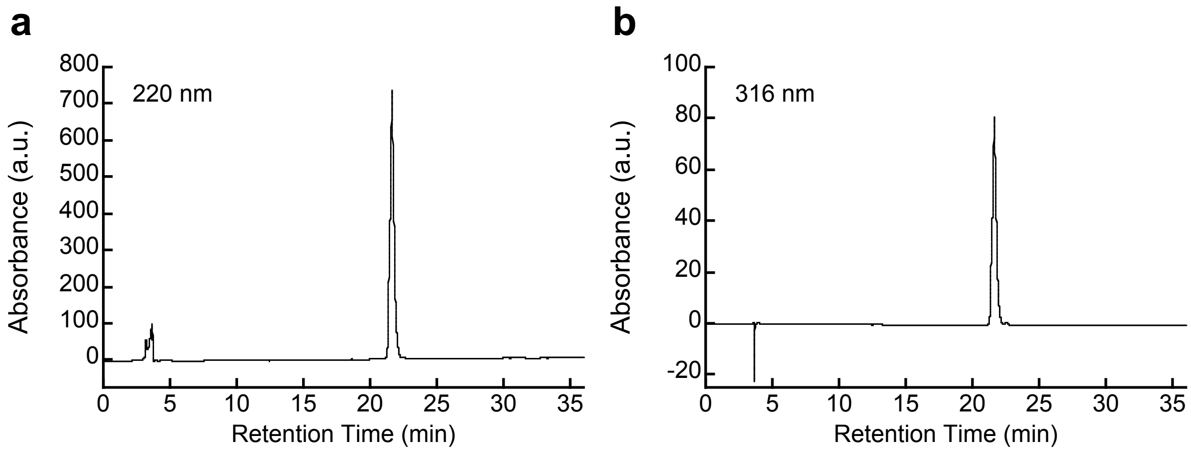

Fig. S29. Analytical HPLC traces of purified DGE-Amx **10** (0% B for 5 min followed by 0-100% B over 30 min, 1 mL/min). (a) Absorbance monitored at 220 nm. (b) Absorbance monitored at 316 nm. The sample was dissolved in 1:1 MeCN/H_2O.

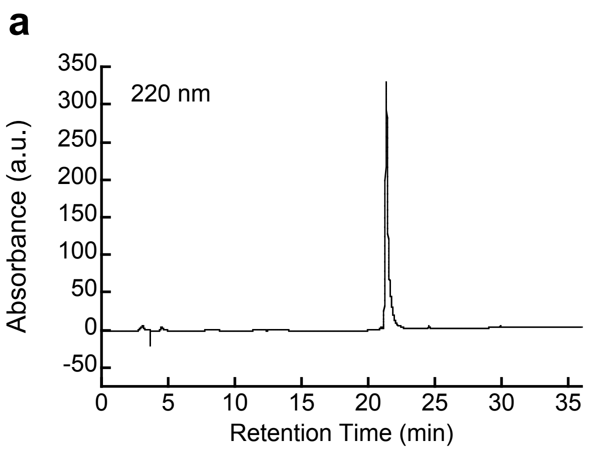

Fig. S30. Analytical HPLC traces of purified MGE-PEG$_3$-N$_3$ **12** (0% B for 5 min followed by 0-100% B over 30 min, 1 mL/min). (a) Absorbance monitored at 220 nm. (b) Absorbance monitored at 316 nm. The sample was dissolved in 1:1 MeCN/H_2O.

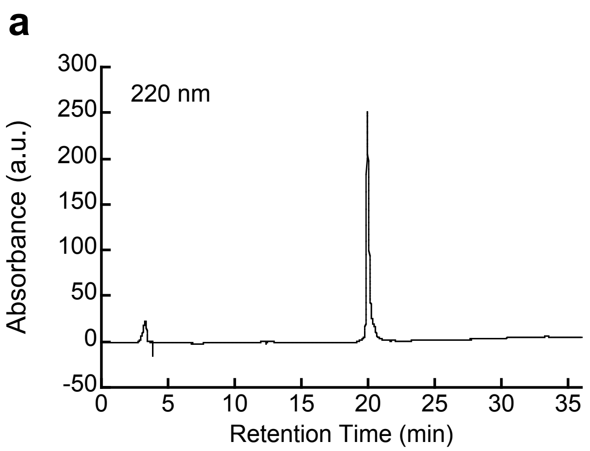

Fig. S31. Analytical HPLC traces of purified DGE-PEG$_3$-N$_3$ **13** (0% B for 5 min followed by 0-100% B over 30 min, 1 mL/min). (a) Absorbance monitored at 220 nm. (b) Absorbance monitored at 316 nm. The sample was dissolved in 1:1 MeCN/H_2O.

Supplementary References

1. T. Zheng and E. M. Nolan, *J. Am. Chem. Soc.*, 2014, **136**, 9677-9691.
2. Z. Zhang, S. Schwartz, L. Wagner and W. Miller, *J. Comput. Biol.*, 2000, **7**, 203-214.
3. T. D. Minogue, H. A. Daligaul, K. W. Davenport, K. A. Bishop-Lilly, S. M. Broomall, D. C. Bruce, P. S. Chain, O. Chertkov, S. R. Coyne, T. Freitas, K. G. Frey, H. S. Gibbons, J. Jaissle, C. L. Redden, C. N. Rosenzweig, Y. Xu and S. L. Jonhson, *Genome Announc.*, 2014, **2**, e00969-14.
4. M. Reister, K. Hoffmeier, N. Krezdorn, B. Rotter, C. Liang, S. Rund, T. Dandekar, U. Sonnenborn and T. A. Oelschlaeger, *J. Biotechnol.*, 2014, **187**, 106-107.
5. R. A. Welch, V. Burland, G. Plunkett III, P. Redford, P. Roesch, D. Rasko, E. L. Buckles, S.-R. Liou, A. Boutin, J. Hackett, D. Stroud, G. F. Mayhew, D. J. Rose, S. Zhou, D. C. Schwartz, N. T. Perna, H. L. T. Mobley, M. S. Donnenberg and F. R. Blattner, *Proc. Natl. Acad. Sci. U. S. A.*, 2002, **99**, 17020-17024.
6. J. Zdziarski, E. Brzuszkiewicz, B. Wullt, H. Liesegang, D. Biran, B. Voigt, J. Grönberg-Hernandez, B. Ragnarsdottir, M. Hecker, E. Z. Ron, R. Daniel, G. Gottschalk, J. Hacker, C. Svanborg and U. Dobrindt, *Plos Pathog.*, 2010, **6**, e1001078.
7. B. Hochhut, C. Wilde, G. Balling, B. Middendorf, U. Dobrindt, E. Brzuszkiewicz, G. Gottschalk, E. Carniel and J. Hacker, *Mol. Microbiol.*, 2006, **61**, 584-595.
8. D. G. Moriel, I. Bertoldi, A. Spagnuolo, S. Marchi, R. Rosini, B. Nesta, I. Pastorello, V. A. Corea, G. Torricelli, E. Cartocci, S. Savino, M. Scarselli, U. Dobrindt, J. Hacker, H. Tettelin, L. J. Tallon, S. Sullivan, L. H. Wieler, C. Ewers, D. Pickard, G. Dougan, M. R. Fontana, R. Rappuoli, M. Pizza and L. Serino, *Proc. Natl. Acad. Sci. U. S. A.*, 2010, **107**, 9072-9077.
9. P. J. Gemski, A. Takeuchi, O. Washington and S. B. Formal, *J. Infect. Dis.*, 1972, **126**, 523-530.
10. F. Yang, J. Yang, X. Zhang, L. Chen, Y. Jiang, Y. Yan, X. Tang, J. Wang, Z. Xiong, J. Dong, Y. Xue, Y. Zhu, X. Xu, L. Sun, S. Chen, H. Nie, J. Peng, J. Xu, Y. Wang, Z. Yuan,

Y. Wen, Z. Yao, Y. Shen, B. Qiang, Y. Hou, J. Yu and Q. Jin, *Nucleic Acids Res.*, 2005, **33**, 6445-6458.

11. D. O. Krause, A. C. Little, S. E. Dowd and C. N. Bernstein, *J. Bacteriol.*, 2011, **193**, 583.
12. S. L. Chen, C.-S. Hung, J. Xu, C. S. Reigstad, V. Magrini, A. Sabo, D. Blasiar, T. Bieri, R. R. Meyer, P. Ozersky, J. R. Armstrong, R. S. Fulton, J. P. Latreille, J. Spieth, T. M. Hooton, E. R. Mardis, S. J. Hultgren and J. I. Gordon, *Proc. Natl. Acad. Sci. U. S. A.*, 2006, **103**, 5977-5982.
13. S. Conlan, P. J. Thomas, C. Deming, M. Park, A. F. Lau, J. P. Dekker, E. S. Snitkin, T. A. Clark, K. Luong, Y. Song, Y.-C. Tsai, M. Boitano, J. Dayal, S. Y. Brooks, B. Schmidt, A. C. Young, J. W. Thomas, G. G. Bouffard, R. W. Blakesley, N. C. S. Program, J. C. Mullikin, J. Korlach, D. K. Henderson, K. Frank, M., T. N. Palmore and J. A. Segre, *Sci. Transl. Med.*, 2014, **6**, 254ra126.
14. C. A. Broberg, W. Wu, J. D. Cavalcoli, V. L. Miller and M. A. Bachman, *Genome Announc.*, 2014, **2**, e00924-14.
15. K. M. Wu, L. H. Li, J. J. Yan, N. Tsao, T. L. Liao, H. C. Tsai, C. P. Fung, H. J. Chen, Y. M. Liu, J. T. Wang, C. T. Fang, S. C. Chang, H. Y. Shu, T. T. Liu, Y. T. Chen, Y. R. Shiau, T. L. Lauderdale, I. J. Su, R. Kirby and S. F. Tsai, *J. Bacteriol.*, 2009, **191**, 4492-4501.
16. S. M. Diene, V. Merhej, M. Henry, A. El Filali, V. Roux, C. Robert, S. Azza, F. Gavory, V. Barbe, B. La Scola, D. Raoult and J. M. Rolain, *Mol. Biol. Evol.*, 2013, **30**, 369-383.
17. S. H. Shin, S. Kim, J. Y. Kim, S. Lee, Y. Um, M. K. Oh, Y. R. Kim, J. Lee and K. S. Yang, *J. Bacteriol.*, 2012, **194**, 2373-2374.
18. X. Didelot, R. Bowden, T. Street, T. Golubchik, C. Spencer, G. McVean, V. Sangal, M. F. Anjum, M. Achtman, D. Falush and P. Donnelly, *PLoS Genet.*, 2011, **7**, e1002191.
